# Overfactoring in rating scale data: A comparison between factor analysis and item response theory

**DOI:** 10.3389/fpsyg.2022.982137

**Published:** 2022-11-30

**Authors:** Javier Revuelta, Carmen Ximénez, Noelia Minaya

**Affiliations:** Department of Psychology, Autonomous University of Madrid, Madrid, Spain

**Keywords:** factor analysis, categorical factor analysis, polychoric correlations, skewness, rating scales, Monte Carlo simulation, item response theory, graded response model

## Abstract

Educational and psychological measurement is typically based on dichotomous variables or rating scales comprising a few ordered categories. When the mean of the observed responses approaches the upper or the lower bound of the scale, the distribution of the data becomes skewed and, if a categorical factor model holds in the population, the Pearson correlation between variables is attenuated. The consequence of this correlation attenuation is that the traditional linear factor model renders an excessive number of factors. This article presents the results of a simulation study investigating the problem of overfactoring and some solutions. We compare five widely known approaches: (1) The maximum-likelihood factor analysis (FA) model for normal data, (2) the categorical factor analysis (FAC) model based on polychoric correlations and maximum likelihood (ML) estimation, (3) the FAC model estimated using a weighted least squares algorithm, (4) the mean corrected chi-square statistic by Satorra–Bentler to handle the lack of normality, and (5) the Samejima’s graded response model (GRM) from item response theory (IRT). Likelihood-ratio chi-square, parallel analysis (PA), and categorical parallel analysis (CPA) are used as goodness-of-fit criteria to estimate the number of factors in the simulation study. Our results indicate that the maximum-likelihood estimation led to overfactoring in the presence of skewed variables both for the linear and categorical factor model. The Satorra–Bentler and GRM constitute the most reliable alternatives to estimate the number of factors.

## Introduction

Ordinal data have an overwhelming presence in educational and psychological measurement (Jöreskog and Moustaki, 2001; Flora and Curran, 2004; Lee et al., 2012). Rating scales, Likert-type items, graded responses, and dichotomous data are the basis for the measurement of attitudes, personality traits, and abilities. By definition, this type of data is bounded between a lower and an upper limit. When the responses concentrate close to one of the boundaries, the distribution of the data presents an extreme mean and either positive or negative skewness. This may happen, for example, when the individuals show a tendency to agree to an opinion scale or when they easily pass the items of an ability test. In some circumstances, the responses are skewed in opposite directions. This pattern of mixed skewness may appear for instance in balanced Likert-type scales containing an equal number of items worded in opposite directions (positive or negative) to control for acquiescence and other response biases (Ferrando and Lorenzo-Seva, 2010; Savalei and Falk, 2014). Usually, a high score in positively worded items is associated to a high factor score whereas the reverse occurs for negatively worded items (Lindwall et al., 2012). Positively and negatively worded items typically have skewness with reversed signs. Another example of mixed skewness occurs when an ability test contains items of varying difficulty to evaluate individuals at different ability levels. Easy items generally have a high mean and negative skewness, and the contrary occurs for difficult items (Ho and Yu, 2015). The same phenomenon may occur with binary data, which is the particular case of rating scales in which responses are scored in two categories.

From a theoretical standpoint, ordinal variables cannot be normally distributed. This is at odds with the assumption of multivariate normality implicit in the most popular methods of statistical inference in the context of factor analysis (FA). However, the assumption of normality is a convenient one from a computational perspective and normal-based methods are still in widespread use under the presupposition that they are reasonable accounts of the data. Alternative methods have been proposed in the psychometric literature to handle non-normal variables (Flora and Curran, 2004). Robust methods are still based on normality and introduce corrections to deal with excess skewness and kurtosis. Models for ordinal data get rid of the normality assumption of manifest variables and explicitly assume a multinomial distribution of responses. There are still no clear answers as to what point one must switch from a normal model to a robust-normal model and to a model for multinomial data.

This article aims to shed some light on this problem by comparing the different inferential methods in a simulation study under conditions of varied skewness. The purpose of the article is to present recommendations to applied practitioners who have rating data and must decide which method to apply from the wide range of ready-to-use options that are available in popular computer programs. The comparison is largely based on Olsson (1979) study, which addressed these effects in relation to the likelihood-ratio chi-square statistic for normally distributed variables. Olsson (1979) investigated several conditions, including sample size, the magnitude of the factor loadings, the number of item response categories, the number of items, and the pattern of skewness. He found that mixed skewness is an important driver for the problem of overfactoring in the context of exploratory FA. Other relevant studies are those by Foldnes and Grønneberg (2022) and Grønneberg and Foldnes (2022), who found that the dissimilarity between the distribution of the latent responses and the assumed normal distribution leads to a distortion in the polychoric correlations and, ultimately, in the results of the categorical factor model.

Olsson (1979) also found that overfactoring is associated with the magnitude of the factor loadings, occurring that higher factor loadings are associated with an increased chi-square value and, consequentially, lead to a worse fit. This phenomenon has been termed the *reliability paradox* (Hancock and Mueller, 2011; McNeish et al., 2018), meaning that high loadings imply that the measurement is more reliable but are associated with an inflated chi-square value and overfactoring. One tentative solution is to switch from FA for normal variables to categorical factor analysis (FAC). Previous research has investigated categorical factor models under different study conditions and estimation methods. For example, Curran et al. (1996) investigated alternative estimation methods and recommended the use of ADF estimation (Browne, 1984) for models including skewed variables. However, ADF is problematic when the sample size includes only a few hundred observations (Hu et al., 1992), which poses a problem for applied investigators in the field of behavioral sciences who typically cannot afford samples of thousands of individuals in their studies.

This article compares five approaches for the FA of ordinal data. First, FA for normal data is considered because it is the first method that was developed (Lawley and Maxwell, 1971) and it is still in widespread use. The second approach is FAC estimated from the polychoric correlation matrix and a maximum-likelihood algorithm (Christoffersson, 1975; Muthén, 1978). The third approach is weighted least squares estimation with mean and variance correction (WLSMV) of the FA model, which has proved to be appropriate for the analysis of ordinal data (Flora and Curran, 2004; Li, 2016). The fourth approach is the mean corrected chi-square by Satorra–Bentler (*T*_*M*_; Satorra and Bentler, 2001, 2010; see also Savalei, 2018), which is a modification of the chi-square statistic of FA to correct for the lack of normality in the data. The *T*_*M*_ rescales the chi-square statistic by an amount that reflects the degree of kurtosis, and it is customarily used with ordinal data. Finally, the fifth approach is the Samejima graded response model (GRM; Samejima, 1969, 2016) from the item response theory (IRT). The FAC and GRM share the basic form of the model (the algebraic relation between latent factors and manifest variables; Takane and de Leeuw, 1987) but differ in the estimation method. The GRM is not estimated from the polychoric correlations but from the individual response patterns using a marginal-likelihood/EM algorithm.

We have assumed that the population follows a FAC model because this approach is possibly the most common in the analysis of ordinal data. However, there is also an active line of research about populations that do not conform to FAC but to a Pearson correlation model (Robitzsch, 2020, 2022). Our simulations cannot help to decide what the population model is. We investigate the performance of the different analytic approaches under the assumption of population FAC.

The article is organized into four sections. Section “Factor analysis models and the effect of categorization” presents the theoretical description of the FA models and the effect of skewness on manifest correlations and overfactoring. The section “Simulation study” describes a simulation study to compare the FA and IRT models under several conditions. A real data example is presented in the section “Real data example” to illustrate these problems in an applied setting. Finally, the article concludes with a discussion and recommendations for applied researchers.

## Factor analysis models and the effect of categorization

### Factor analysis models for continuous and categorical data

The linear factor model (FA) for a vector of variables y* of *J* elements is


(1) $$y^{*}=\Lambda \,\xi +\text{e},$$

where ξ is a vector of latent factor, ***e*** is a vector of random measurement errors^1^, and **Λ** is the matrix of factor loadings. Under the additional assumption of normality, the factors, ξ, follow a standard normal distribution, and the errors, ***e***, are distributed as normal (0, φ), where φ is the standard deviation. In consequence, the distribution of y* is multivariate normal with variance-covariance matrix:


(2) Σ=*ΛΛ′+Ψ,

where **Ψ** is the diagonal variance-covariance matrix of ***e***.

The categorical factor analysis model (FAC) assumes that y* is an unobservable variable and that the manifest responses are obtained from y*through a discretization process (Muthén, 1984; Ferrando and Lorenzo-Seva, 2013). For example, the binary factor model assumes that the response to item *j* is 1 if $y_{j}^{*}$ is sufficiently high and 0 otherwise. More specifically:


(3) $$y_{j}=\left\{ \begin{array}{c} 0,\mathrm{if}\,y_{j}^{*}\leq \tau_{j}\\ 1,\mathrm{if}\,y_{j}^{*}>\tau_{j} \end{array}\right.,$$

where *τ*_*j*_ is a threshold parameter. In general, a model for *K* ordered response categories is based on a discretization process for y* based on *K*-1 threshold parameters.

Mathematically, the skewness of the distribution of the manifest variable, *y*_*j*_, depends on the placement of the thresholds. In the case of dichotomous data, the distribution will be skewed when the threshold parameters have an extreme value. On the contrary, the distribution of responses will be roughly symmetric when the threshold (the population mean of ξ) is close to zero. If the threshold is far away from zero, one of the categories will be more probable than the other. In the general case of an item with *K* response categories, skewness is associated to an uneven location of thresholds around the factor mean.

### The attenuation of correlation

In general, the variance-covariance matrix of ***y***, say **Σ**, will not conform to the structure implied by the linear model of Equation (2). However, Muthén and Kaplan (1985) identified some circumstances where the covariance structure of (2) holds for both y* and *y*. In particular, when a single factor model holds for y* and all the items have the same slope, error variance and thresholds, all correlations between y* are equal. In this case, all the pairs of *y*_*j*_ have the same correlation, $\rho_{y}=a\,\rho_{y^{*}}$, where *a* is the attenuation factor.

**FIGURE 1:**
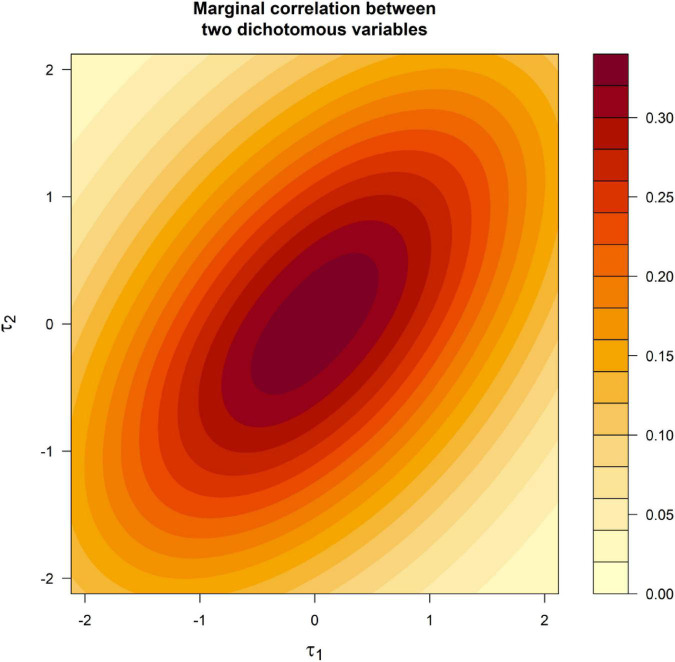
Marginal correlation between the two dichotomous factor indicators as a function of the thresholds.

Figure 1 provides details about the effect of the skewness on the Pearson correlation (Jorgensen and Johnson, 2022), showing the correlation for two variables that measure a common factor. Factor loadings are set to 0.71 and the correlation between *y*_*1*_ and *y*_*2*_ is computed as a function of the threshold.

The most evident result in Figure 1 is that the correlation depends on the threshold and is higher for threshold values close to zero. This is a noteworthy difference between FAC and FA, because in the later model the correlation is mathematically independent of the mean of the variables. A more subtle result shown by Figure 1 is that when the variables are skewed in opposite directions, the attenuation of the correlation is more pronounced and the overfactoring will be potentially more severe. This can be appreciated by comparing the correlations in the corners of Figure 1. For example, the correlations in the corner (−2, −2) are about 0.20 whereas, in the corner (−2, 2), they are about 0.11.

The maximum-likelihood estimation of the FA model consists of finding the values of the parameters that minimize the discrepancy function (Browne and Cudeck, 1992).


(4) F(Σ,Σ)*=log|Σ*|-log|Σ|+trace(ΣΣ*)+p,

where Σ is a variance-covariance matrix and *p* is the number of manifest variables. The population value of the discrepancy is computed by setting **Σ** to the true variance-covariance matrix (for example, those reproduced by a FAC model) and minimizing *F* with respect to Σ*. In estimation, Σ is set to the sample variance-covariance matrix (**Σ** = **S**). The likelihood-ratio chi-square goodness of fit statistic is X2=NF(S,Σ)*, and E(*X*^2^) = *df* (Cudeck and Browne, 1985).

Fitting a FA model to a population that conforms to a FAC model has two unwilling consequences. First, in the special circumstances where the FA fits the true variance-covariance matrix [that is, F(Σ,Σ)*=0 in the population], the FA contains the correct number of latent factors but the slopes will be attenuated. Second, in most cases the FA do not fit the population model [the population value is F(Σ,Σ)*>0]; in that circumstances, the FA cannot reproduce the true variance-covariance matrix, the chi-square has an expectation of E(*X*^2^) = δ + *df* (where δ is a positive non-centrality parameter), and the probability of rejecting the FA model with the correct number of factors will be inflated. The psychometric literature has demonstrated that this bias may lead to distortions such as overfactoring and the emergence of difficulty factors (McDonald and Ahlawat, 1974).

This article compares several widely used approaches to estimate the number of latent factors from ordinal data. These approaches consist of comparing the chi-square associated with a particular model (FA or FAC) and selecting the most parsimonious model that obtains a non-significant statistic. The alternatives are the likelihood-ratio chi-square for the FA model, the modified chi-square statistics that compensate for the lack of normality (Satorra and Bentler, 2010; Bryant and Satorra, 2012), the chi-square associated with FAC under two estimation methods [maximum likelihood (**ML**) and WLSMV], and the (GRM; Samejima, 1969) from IRT. The purpose is to evaluate the precision of the different methods and the potential bias associated with fitting a theoretically incorrect model (the FA). The comparison is conducted *via* simulation studies.

## Simulation study

The present simulation study compares the different approaches for estimating the number of latent factors in exploratory FA when an ordinal factor model with underlying normality holds in the population. Parallel analysis (PA) and categorical parallel analysis (CPA, which is PA applied to polychoric correlations) are also included because of their prominent current use as a method for estimating dimensionality (Lim and Jahng, 2019). Special attention is paid to the differential effect of skewness and magnitude of factor loadings because previous investigations have suggested that these are key conditions in the assessment of model fit (Curran et al., 1996; Ximénez, 2006; Forero and Maydeu-Olivares, 2009).

### Simulated conditions and procedure

The simulated conditions include the sample size (*N*), the number of observed variables (*p*), the number of thresholds (τ), the magnitude of the factor loadings (λ), and the skewness as measured by the γ coefficient (Joanes and Gill, 1998). The levels of these independent variables are summarized in Table 1. In summary, the number of conditions examined was 120 = 2 (sample size levels) × 2 (number of observed variables levels) × 3 (number of thresholds levels) × 2 (loading levels in the factors) × 5 (skewness levels). The number of simulated samples for each condition is 1000.

**TABLE 1:** Conditions of the simulation study.

Code	Variable	Levels
*N*	Sample size	100
		500
*p*	Number of variables	6
		12
τ	Number of thresholds	1 (2 response categories)
		2 (3 response categories)
		4 (5 response categories)
λ	Magnitude of	0.6 (Medium)
	factor loadings	0.9 (High)
γ	skewness	0 (None)
		2 (strong and positive: SP)
		−1 and 1 (Mild and mixed: MM)
		−2 and 2 (Strong and mixed: SM)
		−2, 0 and 2 (None + Strong and mixed: N + SM)

We have manipulated *N* because of its direct relation to chi-square and the potential effect on the results. The number of factors may have an impact on the results of FA given that a model for continuous variables might better approximate the data when the number of response categories increases. The number of variables is manipulated because the size of the fitted model is another condition that affects the population value of the discrepancy function and might influence the results of the chi-square.

The simulating model is a common-factor model (one latent factor) for categorical variables that realistically represents data from rating scales. We have simulated only the one-factor model to keep the number of conditions at a manageable level. Because the true model contains a single factor, we conclude that overfactoring occurs whenever the number of estimated factors is more than one.

The following models were fitted to each sample:

(1) **Factor analysis model fitted by ML**. It was estimated using the *fa* function of the *psych* package in R (Revelle, 2021).
(2) **Categorical factor analysis model**. It is the FAC model estimated using a maximum-likelihood algorithm from the polychoric correlation matrix. It was estimated using the *fa* function of the *psych* package in R (Revelle, 2021).
(3) The FAC model estimated using a weighted least squares estimation method (WLSMV) from the polychoric correlation matrix. The WLSMV method has proved to be appropriate for the analysis of ordinal data (Flora and Curran, 2004; Forero et al., 2009; Li, 2016). This method was applied using the *lavaan* package in R (Rosseel, 2012), which provides a normal-theory likelihood-ratio chi-square statistic used to select the number of latent factors in an exploratory analysis.
(4) The estimator WLSMV in lavaan provides two values of chi-square, the likelihood-ratio chi-square and the scaled chi-square statistic by Satorra and Bentler (2010). The idea of scaling is to correct the distribution of the statistic to match the mean and variance of the theoretical chi-square distribution. The scaling correction applies to differences between non-robust chi-square statistics. The difference between the chi-square values of models with one and two factors is another chi-square. The Satorra-Bentler correction of the chi-square difference is the **d*T*_*M*_ statistic**, which is used to evaluate whether the difference between the one- and two-factor models is significant (Bandalos, 2014; DiStefano and Morgan, 2014). The d*T*_*M*_ was estimated in *lavaan* using the *lavTestLRT* function.
(5) The **GRM model** assumes the same relation between manifest variables and latent factors as the FAC model. The specificity of GRM is that it is estimated using a marginal maximum-likelihood estimation algorithm implemented in the *mirt* package of R (Chalmers, 2012). This package uses a logit link to relate manifest variables and latent factors. The logit link is a convenient mathematical approximation to the normal ogive model, so it is based on the same assumption of normally distributed errors as the FAC model.

The R code for fitting these models appears in Appendix E of the Supplementary materials.

The procedure for simulating categorical data with a prescribed value of skewness is due to Olsson (1979). We are working in the specific case of ordinal data with binomial marginals. The binominal marginal has one free parameter, π, which can be chosen to get the desired skewness. Let γ be the coefficient of skewness and *r* be the number of thresholds. Using Equation (7) in Olsson (1979), the parameter π of the binomial distribution with given values of *r* and γ is:


(5) $$\pi =\frac{1}{2}-\sqrt{\frac{\gamma^{2}\,r}{16+4\,\gamma^{2}\,r}}$$

Once that π has been computed from Equation (5), the second step of the procedure consists of computing the cumulative probabilities of the binomial (*r*, π) distribution, say ***pr*** = [*pr*_1_, *pr*_2_, …, *pr*_(r+1)_]. Finally, the thresholds τ_1_, …, τ_*k*_ are the standard normal deviates associated to the probabilities given by ***pr***; that is, the thresholds are τ_*t*_ = *F*^−1^(*pr*_*t*_) for *t* = 1, …, *r*.

Table 2 shows the threshold values associated with the different values of γ and *r* manipulated in the simulation study. The same thresholds were used for all the variables that have the same value of γ. Notice that it is not guaranteed that the set of thresholds associated with a given γ is unique. Other algorithms may remove the assumption of binomial marginals and render different thresholds, which could have an impact on the results of the simulation. The R code for implementing this procedure is given in Appendix E.

**TABLE 2:** Threshold values for the conditions of the simulation study.

	Number of thresholds						
	
Gamma	1 threshold	2 thresholds		4 thresholds			
			
	τ_1_	τ_1_	τ_2_	τ_1_	τ_2_	τ_3_	τ_4_
-2	–1.05	–2.39	–0.93	–4.32	–3.26	–2.16	–0.86
-1	–0.59	–1.70	–0.31	–3.31	2.28	–1.25	–0.08
0	0.00	–0.67	0.67	–1.53	–0.49	0.49	1.53
1	0.59	0.31	1.70	0.08	1.25	2.28	3.31
2	1.05	0.93	2.39	0.86	2.16	3.26	4.32

In all simulated conditions, the population model is FAC. However, there are conditions where the FA is capable of reproducing the true population variance-covariance matrix and there are others where it is not. As explained in Section “Simulation study,” the FA can reproduce the variance-covariance matrix when all the items have the same parameters (slopes, error variance, and thresholds; that is, in the conditions with no mixed skewness because mixed skewness means that the items have different thresholds). In the conditions with no mixed skewness, the FA and the FAC models are equivalent in the population, the two models have the same number of factors, and attenuation coefficients for the Pearson correlations (Muthén and Kaplan, 1985) take the values shown in Supplementary Table 1. In these cases, any discrepancy between the number of factors estimated by FA and FAC is not attributable to the population model and the simulation provides information about the relative performance of the estimation methods.

In the conditions with mixed skewness (mild and mixed skewness, strong and mixed, none + strong, and mixed), the maximum-likelihood discrepancy function for the FA model attains a nonzero value in the population. The population value of the discrepancy and the RMSEA (Browne and Cudeck, 1992) appear in Supplementary Table 2. The consequence is an inflated chi-square statistic and an increased probability of rejecting a FA model that contains the correct number of factors. In these cases, the simulations inform about the magnitude of the overfactoring effect due to fitting an incorrect model (the FA). Notice that the conditions of mixed skewness are more realistic and representative of practical applications, where the variables rarely are perfectly parallel measures.

The data were analyzed using the empirical proportion of samples (EPS) in which the one-factor model is retained for each condition. The logit of the *p*-value was analyzed as a function of the independent variables manipulated in the study using an ANOVA model to compute the effect size (partial-η^2^) associated with each condition. Notice that the EPS is the proportion of correct decisions, and 1—EPS is the proportion of samples in which the latent dimensionality is overestimated. Thus, the EPS can be interpreted as the statistical specificity of the testing procedure and 1—EPS as the Type-I statistical error.

All the analyses were conducted using the R programming language (R Core Team, 2021) and the aforementioned packages. Some other R packages were used for the analysis of the results: the skewness and kurtosis were estimated using *e1071* (Meyer et al., 2021), and the ANOVA table of the results of the simulation was computed using *sjstats* (Lüdecke, 2021). All the methods used in this article are included in the R language and the indicated packages, so they are readily available and free of charge for any practitioner.

### Results of the simulation

#### Descriptive analysis of the empirical proportion of samples

The EPS for the true one-factor model computed across all simulated conditions is 0.33 for FA, 0.05 for FAC, 0.78 for WLSMV, 0.80 for d*T*_*M*_, and 0.75 for GRM. Regarding parallel analysis, the EPS mean is 0.51 for PA and 0.55 for CPA. This overall result is congruent with the problems of the linear factor model analyzing ordinal data and reflects the improvement with the Satorra-Bentler chi-square and the GRM. These figures are disaggregated now to analyze the effects of the independent variables separately.

Table 3 summarizes the EPS as a function of the skewness (which determines if the FA model is equivalent to the FAC model or not) and the magnitude of the factor loadings in the condition with 6 variables. The results show a strong decrease in the EPS associated with FA and FAC in the conditions of high factor loadings and mixed skewness. However, this decrease does not occur for WLSMV and d*T*_*M*_, which generally provide a good estimation of the number of factors. Interestingly, the polychoric-based methods (CPA, FA, WLSMV, and d*T*_*M*_) do not provide similar results, and the estimation method applied in connection to polychoric correlations apparently is a quite decisive element. The GRM is not as precise as WLSMV or d*T*_*M*_ but it is clearly superior to FA and FAC.

**TABLE 3:** Empirical proportion of samples where the one-factor model is retained depending on the status of the linear model, the skewness, the magnitude of the factor loadings, and the method.

Linear model	Skew	Factor loadings	PA	FA	WLSMV	d*T*_*M*_	CPA	FAC	GRM
True	None	Medium	0.998	0.867	0.961	0.931	0.999	0.431	0.868
		High	0.908	0.708	0.981	0.964	0.712	0.131	0.839
	SP	Medium	0.925	0.629	0.937	0.925	0.955	0.024	0.881
		High	0.546	0.234	0.985	0.977	0.162	0.003	0.869
False	MM	Medium	0.074	0.807	0.954	0.934	0.814	0.160	0.876
		High	0.787	0.004	0.979	0.970	0.447	0.009	0.881
	SM	Medium	0.000	0.545	0.971	0.967	0.818	0.005	0.897
		High	0.143	0.000	0.961	0.961	0.045	0.000	0.998
	None + SM	Medium	0.009	0.760	0.966	0.955	0.731	0.025	0.883
		High	0.582	0.007	0.994	0.994	0.132	0.002	0.916

Results for the conditions with 6 variables. The linear model is true when all the variables have the same slope, error variance and thresholds (conditions of no skewness and strong positive skewness). PA is parallel analysis, CPA is Categorical parallel analysis, FA is normal factor analysis, FAC is categorical factor analysis from the tetrachoric correlation matrix, WLSMV is FA estimated using a robust diagonally weighted least square estimation method, d*T_M_* is the Satorra-Bentler mean corrected chi-square difference, and GRM is the Samejima’s graded response model.

Table 4 shows the PA in relation to skewness and magnitude of the factor loadings in the condition with 12 variables. The results confirm the poor performance of the ML and tetrachoric estimation methods. The WLSMV, d*T*_*M*_, and GRM are stable across all conditions, although the EPS for GRM is not as optimal as in the other two methods. This stability is a crucial result since it means that, under the simulated conditions, the performance of these methods remains constant no matter what the skew of the factor indicators is.

**TABLE 4:** Empirical proportion of samples where the one-factor model is retained depending on the status of the linear model, the skewness, the magnitude of the factor loadings, and the method.

Linear model	Skew	Factor loadings	PA	FA	WLSMV	d*T*_*M*_	CPA	FAC	GRM
True	None	Medium	1.00	0.611	0.910	0.929	1.00	0.084	0.608
		High	0.977	0.348	0.984	0.969	0.682	0.002	0.525
	SP	Medium	0.930	0.096	0.792	0.938	0.962	0.000	0.609
		High	0.535	0.001	0.983	0.987	0.017	0.000	0.582
False	MM	Medium	0.017	0.431	0.863	0.915	0.836	0.002	0.612
		High	0.823	0.000	0.960	0.967	0.334	0.000	0.580
	SM	Medium	0.000	0.167	0.875	0.942	0.720	0.000	0.620
		High	0.292	0.000	0.733	0.948	0.000	0.000	0.798
	None + SM	Medium	0.000	0.323	0.875	0.937	0.707	0.000	0.610
		High	0.578	0.000	0.844	0.997	0.015	0.000	0.640

Results for the conditions with 12 variables. The linear model is true when all the variables have the same slope, error variance and thresholds (conditions of no skewness and strong positive skewness). PA is parallel analysis, CPA is Categorical parallel analysis, FA is normal factor analysis, FAC is categorical factor analysis from the tetrachoric correlation matrix, WLSMV is FA estimated using a robust diagonally weighted least square estimation method, d*T_M_* is the Satorra-Bentler mean corrected chi-square difference, and GRM is the Samejima’s graded response model.

Table 5 contains the PA in relation to skewness and sample size for the conditions with 6 variables. The increase in sample size has a small effect on PA except in the conditions of ML and PA in connection to the FA model.

**TABLE 5:** Empirical proportion of samples where the one-factor model is retained depending on the status of the linear model, the skewness, the sample size, and the method.

Linear model	Skew	*N*	PA	FA	WLSMV	d*T*_*M*_	CPA	FAC	GRM
True	None	100	0.948	0.788	0.971	0.962	0.852	0.276	0.863
		500	0.959	0.787	0.971	0.933	0.859	0.286	0.845
	SP	100	0.707	0.444	0.952	0.952	0.542	0.012	0.879
		500	0.765	0.418	0.963	0.944	0.574	0.015	0.871
False	MM	100	0.480	0.448	0.963	0.958	0.670	0.085	0.895
		500	0.381	0.364	0.967	0.943	0.591	0.084	0.862
	SM	100	0.126	0.393	0.955	0.955	0.494	0.002	0.968
		500	0.017	0.152	0.975	0.971	0.369	0.004	0.927
	None + SM	100	0.333	0.442	0.982	0.980	0.503	0.011	0.915
		500	0.258	0.325	0.977	0.967	0.359	0.016	0.884

Results for the conditions with 6 variables. The linear model is true when all the variables have the same slope, error variance and thresholds (conditions of no skewness and strong positive skewness). PA is parallel analysis, CPA is Categorical parallel analysis, FA is normal factor analysis, FAC is categorical factor analysis from the tetrachoric correlation matrix, WLSMV is FA estimated using a robust diagonally weighted least square estimation method, d*T_M_* is the Satorra-Bentler mean corrected chi-square difference, and GRM is the Samejima’s graded response model.

Table 6 contains the PA in relation to skewness and sample size for the conditions with 12 variables. The pattern of results is similar to Table 6 with a noticeable effect; the EPS for GRM reduces with the increase of the number of variables. This is because the GRM is fitted to the contingency table of the response patterns, which is sparser as the number of variables increases.

**TABLE 6:** Empirical proportion of samples where the one-factor model is retained depending on the status of the linear model, the skewness, the sample size, and the method.

Linear model	Skew	*N*	PA	FA	WLSMV	d*T*_*M*_	CPA	FAC	GRM
True	None	100	0.984	0.502	0.917	0.987	0.834	0.041	0.581
		500	0.993	0.456	0.974	0.911	0.848	0.044	0.552
	SP	100	0.704	0.052	0.782	0.974	0.477	0.000	0.594
		500	0.762	0.045	0.926	0.948	0.502	0.000	0.597
False	MM	100	0.484	0.303	0.849	0.974	0.644	0.001	0.608
		500	0.356	0.128	0.938	0.910	0.526	0.001	0.584
	SM	100	0.287	0.165	0.721	0.941	0.490	0.000	0.731
		500	0.005	0.002	0.792	0.947	0.230	0.000	0.687
	None + SM	100	0.432	0.272	0.757	0.979	0.489	0.000	0.653
		500	0.146	0.051	0.939	0.949	0.233	0.000	0.598

Results for the conditions with 12 variables. The linear model is true when all the variables have the same slope, error variance and thresholds (conditions of no skewness and strong positive skewness). PA is parallel analysis, CPA is Categorical parallel analysis, FA is normal factor analysis, FAC is categorical factor analysis from the tetrachoric correlation matrix, WLSMV is FA estimated using a robust diagonally weighted least square estimation method, d*T_M_* is the Satorra-Bentler mean corrected chi-square difference, and GRM is the Samejima’s graded response model.

The results concerning skewness and the number of thresholds appear in Tables 7, 8 for the conditions with 6 and 12 items, respectively. An increase in the number of thresholds is associated with a mild increase in correct estimations of dimensionality for the FA (in the conditions without mixed skewness) and a reduction for the GRM. One tentative explanation is that more response categories mean that the normal distribution better represents the data and the contingency table of the responses (used for GRM estimation) is sparser. Once again, the WLSMV and d*T*_*M*_ are the best methods, and PA supersedes CPA.

**TABLE 7:** Empirical proportion of samples where the one-factor model is retained depending on the status of the linear model, the skewness, the number of thresholds, and the method.

Linear model	Skew	Thresholds	PA	FA	WLSMV	d*T*_*M*_	CPA	FAC	GRM
True	None	1	0.917	0.657	0.971	0.953	0.715	0.068	0.897
		2	0.966	0.827	0.971	0.947	0.894	0.265	0.861
		4	0.976	0.879	0.971	0.941	0.957	0.510	0.802
	SP	1	0.667	0.400	0.970	0.964	0.503	0.006	0.898
		2	0.753	0.432	0.953	0.942	0.568	0.014	0.868
		4	0.787	0.462	0.948	0.933	0.603	0.021	0.859
False	MM	1	0.357	0.416	0.979	0.971	0.336	0.020	0.921
		2	0.433	0.397	0.963	0.944	0.728	0.082	0.864
		4	0.501	0.405	0.951	0.930	0.828	0.153	0.850
	SM	1	0.037	0.299	0.948	0.948	0.486	0.002	0.960
		2	0.077	0.261	0.979	0.976	0.436	0.002	0.947
		4	0.101	0.258	0.978	0.973	0.372	0.005	0.936
	None + SM	1	0.220	0.400	0.979	0.977	0.302	0.005	0.925
		2	0.318	0.377	0.983	0.974	0.486	0.011	0.909
		4	0.347	0.373	0.975	0.967	0.504	0.024	0.865

Results for the conditions with 6 variables. The linear model is true when all the variables have the same slope, error variance and thresholds (conditions of no skewness and strong positive skewness). PA is parallel analysis, CPA is Categorical parallel analysis, FA is normal factor analysis, FAC is categorical factor analysis from the tetrachoric correlation matrix, WLSMV is FA estimated using a robust diagonally weighted least square estimation method, d*T_M_* is the Satorra-Bentler mean corrected chi-square difference, and GRM is the Samejima’s graded response model.

**TABLE 8:** Empirical proportion of samples where the one-factor model is retained depending on the status of the linear model, the skewness, the number of thresholds, and the method.

Linear model	Skew	Thresholds	PA	FA	WLSMV	d*T*_*M*_	CPA	FAC	GRM
True	None	1	0.973	0.283	0.932	0.958	0.615	0.000	0.639
		2	0.996	0.502	0.957	0.948	0.920	0.009	0.593
		4	0.996	0.652	0.949	0.938	0.988	0.119	0.468
	SP	1	0.659	0.047	0.897	0.979	0.462	0.000	0.627
		2	0.754	0.051	0.852	0.944	0.496	0.000	0.592
		4	0.785	0.048	0.841	0.933	0.510	0.000	0.567
False	MM	1	0.407	0.229	0.914	0.963	0.271	0.000	0.666
		2	0.400	0.206	0.886	0.918	0.656	0.000	0.565
		4	0.454	0.212	0.902	0.912	0.829	0.002	0.556
	SM	1	0.148	0.094	0.590	0.945	0.430	0.000	0.759
		2	0.147	0.083	0.909	0.955	0.354	0.000	0.700
		4	0.143	0.075	0.885	0.933	0.296	0.000	0.669
	None + SM	1	0.307	0.188	0.795	0.982	0.306	0.000	0.689
		2	0.283	0.152	0.914	0.955	0.394	0.000	0.626
		4	0.277	0.144	0.900	0.939	0.384	0.000	0.561

Results for the conditions with 12 variables. The linear model is true when all the variables have the same slope, error variance and thresholds (conditions of no skewness and strong positive skewness). PA is parallel analysis, CPA is Categorical parallel analysis, FA is normal factor analysis, FAC is categorical factor analysis from the tetrachoric correlation matrix, WLSMV is FA estimated using a robust diagonally weighted least square estimation method, d*T_M_* is the Satorra-Bentler mean corrected chi-square difference, and GRM is the Samejima’s graded response model.

#### ANOVA model and effect size for the logit of *p*-value

An *ANOVA* model was fitted to the logit of the chi-square *p*-value with the purpose of analyzing the interaction between the conditions of the simulation and evaluating the importance of the different independent variables manipulated in the study. The magnitude of the effects was evaluated using the partial-η^2^ as a measure of effect size. The statistical significance is unimportant here as it depends on the number of simulated samples and is easily manipulated.

Supplementary Tables 3–7 summarize the *ANOVA* results. The tables are computed separately for each model and for the conditions with 6 and 12-factor indicators. Regarding the FA model (see Supplementary Table 3), the only interaction that appears to have some importance is λ × γ, especially in the condition with a smaller number of variables. The most relevant main effects are those of factor loading and skewness. Plots of the *p*-value for the FA model appear in Supplementary Figure 1. The *p*-value is closer to zero for the model with 12 variables, resulting in a decreased EPS and smaller interactions because the lines in the plots are mostly flat. The effect of the increase of the factor loadings consists of pulling the *p*-value toward zero as skewness increases.

The same analysis in relation to FAC appears in Supplementary Table 4 and Supplementary Figure 2. In this case, the number of thresholds has a more important role regarding the main effects and the interactions as compared with the FA model, although the effect sizes are smaller because the *p*-value is generally close to zero for all conditions.

The results for WLSMV and d*T*_*M*_ are summarized in Supplementary Tables 5, 6. No relevant effects appear for the short number of variables, which is an excellent result because it means that the performance of these methods is good regardless of the simulated conditions. Some relevant effect sizes appear in conditions with a larger number of variables, but the methods remain reliable also in these conditions.

The results for the GRM appear in Supplementary Table 7 and Supplementary Figure 3. Only the skewness and its interaction with factor loading have a relevant effect size, and only in the condition of the short number of variables. Supplementary Figure 3 shows that the condition of strong and mixed skewness pulls the *p*-value toward 1 but only in the condition of high factor loadings. In any case, this result does not alter the EPS because the *p*-values remain in the acceptance region.

### Recovery of the parameters

The success of a method cannot be based solely on the estimation of the number of factors. A method that correctly estimates the latent dimensionality whilst producing wildly incorrect parameter estimates is of limited usefulness. Parameter estimates under the different models were analyzed to assess their relative merit. Recovery was evaluated by the root mean squared error between the true and estimated parameters (RMSE).

The estimated parameters for FA have two specific sources of error that are not present in the other methods: Attenuation and structural error. As explained previously, attenuation occurs when a FA model is able to reproduce the true variance-covariance parameters (which conform to a FAC model) but the FA parameters have smaller values than the true FAC model. Attenuation occurs in conditions with no skewness and strong positive skewness, and its magnitude depends on the coefficients shown in Supplementary Table 1. In the conditions where the FA cannot reproduce the population variance-covariance matrix of the FAC model (those that involve mixed skewness), the FA estimates have the additional bias of structural error. These are population biases that will not vanish no matter what the sampling size is. The simulations are informative about the magnitude of the three sources of error combined (sampling error, attenuation, and structural error), and the increase of error that is specific to FA in comparison to the other methods.

The *mirt* function estimates the GRM using the IRT parameterization, whereas the true parameter values for the simulation are in the FA parameterization. Previously to computing the RMSE, the IRT parameters were converted to the FA parameterization using the equations (A15) and (A16) of Paek et al. (2018).

The recovery of lambda in relation to the manipulated conditions appears in Supplementary Table 8 and Supplementary Figures 4–6. WLSMV and FAC performed similarly and obtained the higher RMSE in the conditions of a large number of thresholds, small loadings, and strong positive skewness. The results indicate that GRM outperforms these methods and has a smaller RMSE that remains stable across conditions. The FA is affected by attenuation and inconsistency biases and is generally poorer than the other methods. The results for ψ follow a similar pattern and are summarized in Supplementary Table 9.

Recovery of τ is summarized in Supplementary Table 10 and Supplementary Figures 7–9. The results are similar to those found for the recovery of λ. There are small differences between WLSMV and FAC, and the two methods render an increased RMSE in the conditions of a large number of thresholds and strong positive skewness. The GRM obtains a smaller RMSE than the other methods and is stable across conditions.

### Conclusions of the simulation study

The results of our simulation have shown that, in the present context, the chi-square statistic for FA leads to overfactoring. However, there are remarkable differences between the models and the study conditions. The magnitude of the factor loadings and the skewness of the factor indicators have the largest effect size in relation to the logit of the *p*-value, but this effect varies between the models. The empirical proportion of rejection for the one-factor FA model is generally high and sharply increases in the conditions where this model is wrong in the population. The PA shows better performance than the chi-square, but the overfactoring is still high in the presence of skewness. Notice that the effect of skewness might depend on the choice of thresholds values. We have used the algorithm by Olsson (1979) to generate the threshold values associated with the gamma coefficient. However, other algorithms may render different thresholds associated with the same gamma and might have a different effect on the performance of the chi-square. One topic for further investigation would be the development of other algorithms for generating thresholds and the study of the differential impact of the choice of thresholds while keeping gamma fixed to a constant value. The pattern of the factor loadings also has a noticeable effect, and smaller rejection rates are found for the tau-equivalent than for the congeneric model.

Interestingly, dimensionality recovery for the FAC model is tightly related to the estimation method, as recovery is poor for ML and reliable for WLSMV and d*T*_*M*_.

The effect sizes of all independent variables are negligible for the GRM, which indicates that the model is robust to the manipulated conditions. The Type-I error is about 30%, regardless of skewness, which is still far from the nominal levels, but it improves the results of the other models. The number of factor indicators also has an effect, and more rejections of a correct model are found with a larger number of factor indicators. This is an expected result because bigger samples are necessary for the likelihood-ration statistic to approximate its chi-square reference distribution when there are a large number of factor indicators. On the counter side, the small effect size associated with *N* means that the improvement in EPS decreases as *N* increases. From a theoretical point of view, the GRM is not exactly true in this simulation because data were generated assuming a normal distribution for the error (normal link) whereas the implementation of the GRM in the *mirt* package assumes a logit link. The logit link was developed as a convenient approximation to the normal link and the difference between them is small. However, this difference does not vanish as sample size increases, which converts the logit link to an inconsistent procedure. One problem for future research is to investigate if the results of the GRM improve when estimated with a normal link.

Finally, the WLSMV and the Satorra-Bentler d*T*_*M*_ are the best alternatives to estimate dimensionality, with rejection rates close to the nominal 5% level that remains relatively constant across all the simulated conditions. The recovery of parameters for GRM was better than for WLSML, and less affected by those conditions in which the RMSE increases for WLSMV. Thus, a good combination of methods could be to estimate the dimensionality using a Satorra-Bentler fit statistic, as in WLSMV, and estimate parameters using a marginal ML algorithm as in the GRM once the dimensionality of the model has been fixed.

## Real data example

### Data and procedure

A real data sample was analyzed to illustrate the different approaches in an applied context. The data consists of responses to the scale of attitudes toward censorship, originally published by Rosander and Thurstone (1931), and reprinted by Shaw and Wright (1967). The scale is composed of 20 Likert-type items with six response alternatives. The responses are labeled: *Strongly Disagree*, *Disagree*, *Slightly Disagree*, *Slightly Agree*, *Agree*, and *Strongly Agree*. The sample size is 223 observations. The items and the data are publicly available in the Georgia Tech Psychometric Research and Development Lab^2^ and were retrieved on 03/17/2022. We have applied the same methods used in the simulation study (FA, FAC, WLSMV, d*T*_*M*_, and GRM) and some new methods for completeness. The new methods are:

• Descriptive statistics about the type-3 coefficient of skewness and kurtosis are run using the *e1071* package (Meyer et al., 2021). Multivariate coefficients of skewness and kurtosis were computed using the *semTools* package (Jorgensen et al., 2022).
• Mean corrected chi-square, *T*_*M*_ (Satorra and Bentler, 2001) and mean and variance corrected chi-square, *T*_*MV*_ (Asparouhov and Muthén, 2010) computed with the maximum-likelihood estimator for the FA model. The *T*_*M*_ and the *T*_*MV*_ are modified chi-square statistics that evaluate the fit for a single model and correct for the lack of normality in the data. The *T*_*M*_ and the *T*_*MV*_ can be obtained in *lavaan* using the function *cfa* with the arguments *estimator = “MLM”* and *estimator = “MLMV,”* respectively.
• The *T*_*M*_ and *T*_*MV*_ statistics are also computed with WLSMV estimator and FAC model using the arguments *estimator = “WLSMV”* and *ordered = TRUE* to the *cfa* function in *lavaan*. The *T*_*M*_ and the *T*_*MV*_ are not to be confused with the correction of the chi-square difference, d*T*_*M*_, used in the simulation study.
• The analysis of the normality of the latent responses (variable y* in Equation 3) was evaluated using a bootstrap test implemented in the *bootTest* function in the R package *discnorm* (Foldnes and Grønneberg, 2020).
• The analysis of normality of the latent factor (that is, testing that *f*(ξ)is a normal distribution) was run using the empirical histogram method (Woods, 2007) and the Davidian Curve method (Woods and Lin, 2009; Smits et al., 2020). These methods are implemented in the *mirt* function using the arguments *dentype = “empiricalhist*,” and *dentype* = “*Davidian*-#” argument. We have estimated the one-factor FAC model with a normal distribution for *f*(ξ), a Davidian-curve estimate for *f*(ξ) with two smooth parameters, and a Davidian curve with three smooth parameters. The models were compared using the Hannan–Quinn criterion (HQ; Woods, 2006) implemented in the *anova* method of the *mirt* package.
• Multidimensional Nominal Categories Model (MNCN; Revuelta, 2014; Revuelta et al., 2020, 2021). The MNCM is a generalization of the GRM that removes the assumption that the responses follow an ordinal scale. The comparison between the MCNM and GRM provides a means to test the assumption of equally spaced categories implicit in the GRM.

The R codes for all the analyses appear in Appendix G.

In the simulation study, we have assumed that the population model is an ordinal factor model and we have manipulated the parameters of the model to investigate the performance of several estimation methods, but the correctness of the model has not been put into question. In a real data analysis, the validity of an ordinal factor model cannot be taken for granted as it might not be correct in the population, which introduces another source of bias in the analysis. Some of the above analyses (normality of latent responses, normality of latent factors, and the MNCM) evaluate part of the assumptions of the ordinal factor model to determine if they may be maintained for the present sample.

### Results of the empirical study

Table 9 contains the descriptive statistics for the items, including the response frequency of the categories, the mean, the standard deviation, and the kurtosis. The variables have a pattern of mild and mixed skewness. Most of them have a mean value close to the midpoint of the scale, and a skewness value close to zero. However, there are also several variables negatively skewed because of the mean being close to the higher point of the response scale, and one of the variables has a low mean and positive skew. The multivariate skewness and kurtosis coefficient have a *p*-value smaller than 0.01 and the hypothesis of normality is rejected.

**TABLE 9:** Descriptive statistics for the scale of attitudes about censorship.

Item	Freq 1	Freq 2	Freq 3	Freq 4	Freq 5	Freq 6	Mean	Std	skewness	Kurtosis
1	20	35	56	45	33	34	3.62	1.52	0.04	–0.98
2	4	2	12	40	52	113	5.12	1.12	–1.38	1.85
3	31	36	32	61	35	28	3.52	1.58	–0.10	–1.06
4	17	24	39	73	43	27	3.82	1.39	–0.32	–0.57
5	74	61	35	31	13	9	2.44	1.43	0.81	–0.27
6	38	52	57	32	26	18	3.04	1.50	0.40	–0.80
7	40	40	53	47	28	15	3.13	1.49	0.18	–0.91
8	7	8	31	58	56	63	4.51	1.30	–0.67	–0.09
9	92	43	40	25	15	8	2.34	1.45	0.85	–0.31
10	93	63	27	21	14	5	2.17	1.35	1.08	0.25
11	32	42	58	34	36	21	3.28	1.53	0.18	–0.99
12	20	32	39	39	47	46	3.89	1.61	–0.25	–1.12
13	25	29	37	62	44	26	3.67	1.50	–0.24	–0.88
14	10	17	17	75	60	44	4.30	1.33	–0.71	0.03
15	25	26	29	65	48	30	3.78	1.52	–0.37	–0.83
16	12	17	31	81	51	31	4.05	1.31	–0.51	–0.16
17	3	7	14	42	70	87	4.93	1.15	–1.13	0.99
18	40	40	27	69	32	15	3.26	1.53	–0.05	–1.09
19	24	15	21	96	44	23	3.85	1.39	–0.62	–0.18
20	4	5	16	52	65	81	4.85	1.16	–1.00	0.81

The hypothesis of normality of the latent responses is discarded (*p*-value < 0.01). This result threats the validity of the methods based on polychoric correlations since these correlations are highly sensitive to the validity of the normality assumption for the latent responses (Foldnes and Grønneberg, 2020, 2022). However, as we do not have information about what the distribution of the latent responses might be, we are not in a position of estimating polychoric correlations under a different distribution.

Regarding the analysis of normality for the latent factor, the HQ for the normal, Davidian-2, Davidian-3, and empirical histogram models are 13782.5, 13789.3, 13788.2, and 14175.1, respectively. Similar to AIC and BIC, the HQ supports the model that minimizes it. Since the normal distribution minimizes the HQ, the Gaussian distribution can be retained.

The goodness-of-fit statistics for the FA models with one to four factors appear in Table 10, including the chi-square against a saturated model and the modified chi-squares: *T*_*M*_ and *T*_*MV*_. The chi-square estimates three factors whereas the robust statistics estimates two factors. Since the data show non-negligible skewness and kurtosis, these results point in the direction of relying in robust statistics and retaining two factors. Parallel analysis gives a similar result and the number of estimated factors is two for PA and three for CPA.

**TABLE 10:** Chi-square and modified chi-square for the FA model.

	Chi-square			Mean corrected			Mean and variance corrected			
			
Factors	*G* ^2^	*df*	*p*	*T*_*M*_	*c*	*p*	*T*_*MV*_	*a*	*b*	*p*
1	466.2	170	0.00	405.5	1.15	0.00	301.4	2.06	75.18	0.00
2	269.3	151	0.00	**241.0**	**1.12**	**0.00**	**206.8**	**1.80**	**57.42**	**0.00**
3	**194.76**	**133**	**0.00**	174.5	1.12	0.01	160.5	1.69	45.03	0.05
4	151.9	116	0.01	133.5	1.14	0.13	127.8	1.69	38.08	0.21

*G*^2^ is the likelihood ratio chi-square statistic against a saturated model under the assumption of multivariate normality, *df* is the degrees of freedom, *p* is the *p*-value. *T_M_* is the mean corrected *G*^2^ by Satorra and Bentler. *T_MV_* is the mean and variance corrected *G*^2^ by Asparouhov and Muthén. Boldface values indicate the simplest non-rejected model using a nominal type-I error rate of 0.01.

Table 11 contains the goodness-of-fit statistics for FAC with the WLSMV estimator, the chi-square statistics corrected for non-normality (the *T*_*M*_, the *T*_*MV*_), and the Satorra-Bentler correction of the chi-square difference (d*T*_*M*_). The statistics differ in their conclusions. The chi-square and the chi-square difference give support to a three-factor model. On the other hand, the modified chi-square statistics (*T*_*M*_, *T*_*MV*_, and d*T*_*M*_) suggest models with higher dimensionality.

**TABLE 11:** Chi-square and modified chi-square for the FAC model using WLSMV estimation.

Factors	*G* ^2^	*df*	*p*	dif-*G*^2^	dif-*df*	*p*	*T*_*M*_	*p*	*T*_*MV*_	*p*	d*T*_*M*_	*p*
1	642.6	170	0.00				1057.6	0.00	681.8	0.00		
2	211.3	151	0.00	431.4	19	0.00	434.6	0.00	331.9	0.00	128.0	0.00
3	**125.0**	**133**	**0.68**	**86.3**	**18**	**0.00**	287.2	0.00	237.1	0.00	57.4	0.00
4	89.6	116	1.00	35.4	17	0.01	223.5	0.00	190.4	0.00	50.51	0.00
5	61.6	100	1.00	28.0	16	0.03	166.3	0.00	**148.4**	**0.00**	260.3	0.00
6	44.3	85	1.00	17.3	15	0.30	**127.0**	**0.00**	116.3	0.01	65.5	0.00
7	30.7	71	1.00	13.6	14	0.48	93.3	0.04	88.4	0.08	54.7	0.00

*G*^2^ is the likelihood ratio statistic against a saturated model, *df* is the degrees of freedom and p is the *p*-value. dif-*G*^2^ is the difference between any two consecutive values of *G*^2^. *T_M_* is the mean corrected chi-square by Satorra and Bentler. *T_MV_* is the mean and variance corrected chi-square by Asparouhov and Muthén. *T_d_* is the mean corrected Satorra-Bentler statistic applied to the difference between two consecutive values of *G*^2^. Boldface values indicate the simplest non-rejected model using a nominal type-I error rate of 0.01.

The FAC model under ML results in five or six estimated factors, depending on the statistic used to test model fit (chi-square of absolute model testing or chi-square between pairs of nested models). The results appear in Table 12. However, the FAC method based on ML apparently is questionable in light of the simulation results.

**TABLE 12:** Chi-square for the FAC model estimated by maximum likelihood.

Factors	*G* ^2^	*df*	*p*	dif-*G*^2^	*df-dif*	*p-dif*
1	623.7	170	0.00			
2	270.9	151	0.00	253.7	19	0.00
3	370.0	133	0.00	99.2	18	0.00
4	219.5	116	0.00	51.4	17	0.00
5	**163.0**	**100**	**0.00**	56.5	16	0.00
6	121.9	85	0.01	**41.1**	**15**	**0.00**
7	93.1	71	0.04	28.8	14	0.01

*G*^2^ is the likelihood ratio chi-square statistic against a saturated model, *df* is the degrees of freedom, *p* is the *p*-value, dif-*G*^2^ is the likelihood-ratio chi-square for pairs of consecutive models. Boldface values indicate the simplest non-rejected model using a nominal type-I error rate of 0.01.

The MNCM was estimated to evaluate the assumption that responses follow an ordinal scale. The difference between an ordinal and a nominal model is that there is only one slope for each item in the former model and one slope for each response category in the later model (except for one of the categories of the item, which has a slope of zero for identifiability). The results appear in Table 13 and indicate that the MNCM does not significantly fit better than the GRM. Thus the ordinality assumption involved in the GRM can be maintained in light of this analysis.

**TABLE 13:** Chi-square for the GRM and the MNCM models.

	GRM					MNCM					GRM vs. MNCM		
			
Factors	pars.	*LK*	dif-*G*^2^	*df*	*p*	pars.	*LK*	dif-*G*^2^	*df*	*p*	dif-*G*^2^-M	*df*	*p*
1	120	−6688.7				200	−6658.1				61.3	80	0.94
2	139	−6573.7	230.1	19	0.00	219	−6529.2	257.7	19	0.00	88.9	80	0.23
3	**157**	−**6530.5**	**86.3**	**18**	**0.00**	237	−6500.8	56.7	18	0.00	59.3	80	0.96
4	174	−6514.1	32.9	17	0.01	254	−**6482.3**	**37.1**	**17**	**0.00**	63.5	80	0.91
5	190	−6508.4	11.2	16	0.79	270	−6482.2	0.2	16	1.00	52.5	80	0.99

pars. is the number of estimated parameters for each model, LK is the log likelihood, dif-*G*^2^ is the likelihood-ratio chi-square for pairs of consecutive models, df is the degrees of freedom, p is the p-value. dif-*G*^2^-M is the likelihood ratio chi-square of the GRM against the MNCM with the same number of factors. Boldface values indicate the simplest non-rejected model using a nominal type-I error rate of 0.01.

This empirical example shows mixed results. The most parsimonious results are those of FA and GRM, whereas the modified chi-squares lead to models of higher dimensionality. The large number of factors resulting from polychoric-based methods (FAC in its different variants) may be due to an unprecise estimation of polychoric correlations because of the lack of normality of the latent responses. In the simulation, we considered only the case in which latent normality holds. This assumption is not tenable in the empirical application, and the psychometric literature shows that this might be a source of imprecision in the estimates of polychoric correlations (Grønneberg and Foldnes, 2022). All in all, we would recommend the solution with a small number of factors for parsimony and the questionability of polychoric-based methods in this sample.

## Discussion and conclusion

The purpose of this article was to investigate the behavior of the chi-square value of the FA when the data are categorical. We compare different approaches to deal with this problem and examine their performance to determine the number of factors under several design conditions in a simulation study. The article is oriented to investigators analyzing ordinal data, for example from Likert-type items, rating scales, etc. In this respect, we assume that an ordinal factor model is correct in the population and analyze the data using the most common methods available nowadays. It is important to notice that the FA (based on the normality assumption) cannot be generally correct in this situation, and it is included here mainly because of its popularity in the applied field and to illustrate some of the biases that it might produce. Appropriate alternatives to normal-FA that can be easily implemented are the FAC based on WLSMV estimation, the modified chi-square statistics, and the GRM.

Our results concur with those of the psychometric literature, pointing out two important drivers for overfactoring related to FA: the magnitude of factor loadings and the data skewness. We have stressed the importance of skewness because this phenomenon is inherent to ordinal data and perhaps has not received as much attention in the literature as the issue of factor loadings. More specifically, mixed skewness is of particular importance due to its effect on attenuating the correlation between factor indicators and the subsequent increase in the number of estimated factors. The results of our simulation study are congruent with Olsson (1979)’s study and provide new insights into the performance of the robust methods and the FA for categorical variables.

Ordinal data generally present skewness because these data come from bounded response formats, such as Likert and rating scales, which are the response formats most commonly used in educational and psychological responses. Bounded scales generate asymmetric distributions when the mean of the variable approaches the lower or the upper bound. The FA model based on the normality assumption is often applied routinely to these data albeit being incorrect, which results in overfactoring and inconsistent parameter estimates. Our results show that mixed skewness and high factor loadings are especially associated to overfactoring in connection with this method. The other conditions manipulated in the simulation (including sample size, number of factor indicators, and number of response categories) were of secondary importance given the simulated conditions.

The problems with the FA model are not surprising since they have been documented for quite some time in the psychometric literature (Muthén and Kaplan, 1985). However, our results showed that the factor model based on polychoric correlations (the FAC model) can be problematic if the estimation algorithm is not selected carefully. In general, the estimation operates in two stages: (1) Estimate the polychoric correlations from the categorical responses, and (2) estimate factor parameters from the polychoric correlations (using either maximum-likelihood or WLSMV in our simulations). The results show that the choice of an estimation method in stage 2 has important consequences, and WLSMV is far superior to any other method. However, the conclusions of the simulation depend on the conditions used in our study, which are ordinal data with binomial marginals and exact underlying normality. Some aspects in which our simulations can be extended are to consider other estimation methods, larger sample sizes, and different patterns of item thresholds. Apart from this, the estimation of polychoric correlations is sensitive to the normality assumption of the latent responses (Foldnes and Grønneberg, 2020), which is another area of active investigation.

The GRM model from IRT has shown better performance than FAC albeit being based on similar assumptions. One important advantage of GRM over FAC is that it avoids the use of polychoric correlations by relying on a marginal ML estimation procedure (MMLE; Bock and Aitkin, 1981). However, the Type-I error rate for the GRM is about 30%, still far from the nominal 5% level. GRM has two inherent limitations, the numerical integration procedure involved in estimation (the so-called *curse of dimensionality*) and the sparseness of the contingency table of manifest responses when the number of variables increases. MMLE cannot be applied with more than five or six factors. However, some recent advances (Cai, 2010) have opened the way to apply it with a larger number of factors, and constitute a viable alternative to factor-analyzing ordinal data.

All in all, our results suggest that the most tenable method for estimating dimensionality is the family of modified chi-square statistics (*T*_*M*_, *T*_*MV*_, and *T*_*d*_) in connection with the WLSMV estimation method, as long as the assumption of underlying normality can be maintained. The Type-I error rate is about the nominal level for all conditions, which is also a very convenient property because researchers do not have to worry about which statistic to use and in which circumstances. However, the simulation also shows that recovery of the parameters is more precise for GRM than for the other methods. One possible explanation for this phenomenon is that GRM is estimated by a full-information marginal ML procedure that fits the model to the individual response patterns, whereas the polychoric-based methods use a limited information estimation method that fits the model to the second-order moments.

Parallel analysis is nowadays widely used to estimate the number of factors due to the problems associated with chi-square. The results of our simulation show that PA (based on Pearson correlations) performs better than FA but not better than GRM or *T*_*d*_. The parallel analysis for categorical data inherited the problems associated with polychoric correlations and overfactoring persisted.

Apart from the aforementioned extensions of our simulated conditions, there are other directions in which this research can be expanded. One is to investigate other goodness-of-fit indices not considered here. Drawing on recent research by Ximénez et al. (2022), future research could be directed to assess the performance of the RMSEA and the unbiased SRMR index, which are consistent and asymptotically unbiased estimators of the parameter of interest and have shown good statistical properties and efficiency to provide interpretation guidelines to assess the goodness-of-fit (Shi et al., 2018; Ximénez et al., 2022).

Future studies may also examine the consequences of distributional assumptions at different levels (Zygmont and Smith, 2014), which is currently a field of active psychometric research. Regarding manifest variables, the present article focuses on raking data because of the widespread use of Likert-type items in psychological and educational measurement. However, other types of variables may require a specific model. For example, continuous bounded variables may be analyzed with a factor model based on the beta distribution (Revuelta et al., 2022). A FA model can also be based on manifest indicators measured on a nominal scale (Revuelta et al., 2020, 2021).

The methods for categorical data evaluated in this article are based on the assumption that manifest responses are the result of a discretization process for a latent response, where both latent factors and errors follow a normal distribution. This currently is the common setup in applied psychometrics, and this article is oriented to practitioners that analyze ordinal data with the common methods. However, there is also an active line of research that questions the normality of factors and errors (Grønneberg and Foldnes, 2022; Jobst et al., 2022; Manapat and Edwards, 2022). The validity of the conclusion from our simulations is contingent upon the normality and the other assumptions involved in the categorical factor model. While the literature has shown that tetrachoric correlations are distorted by violations of normality (Grønneberg et al., 2020), more investigation is needed to determine the consequences of this violation in the other methods, as well as the estimation of the distribution of latent responses (Foldnes and Grønneberg, 2020, 2022). In the meantime, if the investigator insists on using polychoric-based methods, normality tests for latent responses shall be run before applying these methods.

Another generalization of the proposed methods consists of estimating the distribution of the latent factor instead of assuming a Gaussian variable. The present article has applied several options for the one-dimensional latent space (Woods, 2006; Woods and Lin, 2009), but extensions of these methods for multiple-factor models do not exist yet. One final area of investigation is the nominal factor model, which assumes that latent variables are Gaussian although it includes one latent response for each category instead of one response for the complete item as in the FAC model (Revuelta, 2014; Revuelta et al., 2021). The nominal factor model is more flexible and can fit the data in some circumstances where the FAC cannot. The development of intermediate models between the full nominal model and the FAC is another way of defining models that better represent the data and achieve an acceptable fit (Thissen et al., 2010).

## Data availability statement

Publicly available datasets were analyzed in this study. This data can be found here: https://prdlab.gatech.edu/unfolding/data/.

## Author contributions

JR contributed to the conception of the manuscript, the design and planning of the simulation study, the development of the R code for the data simulation, the statistical analysis and interpretation of results, and the drafting of the manuscript. CX participated in the literature review, implementation of the robust statistical procedures, statistical analysis, and the revision of the manuscript. NM contributed to the implementation of the simulation study and the data analyses. All authors contributed to the article and approved the submitted version.

## Footnotes

1 In the original formulation of the model, ***e*** splits into a unique factor and a measurement error. However, such a distinction is mainly of a theoretical interest as these two elements cannot be empirically distinguished in most practical settings.

2
https://prdlab.gatech.edu/unfolding/data/
