# Supplementary material for: Overfactoring in rating scale data: A comparison between factor analysis and item response theory
Source: Front Psychol. 2022 Nov 30;13:982137. doi: 10.3389/fpsyg.2022.982137 (PMC9750161; doi:10.3389/fpsyg.2022.982137)
Supplement: Supplementary file 1 [file Data_Sheet_1.docx]

**Supplementary material for**

**Overfactoring in rating scale data: A comparison between factor analysis and item response theory**

Javier Revuelta

Carmen Ximénez

Noelia Minaya

**APPENDIX A. Population fit when the FA model applies to CFA data**

**Table S1.**

*Attenuation factor in the population in the conditions when the maximum-likelihood discrepancy function is zero*

|  | thresholds | *λ* = 0.60 | *λ* = 0.90 |
| --- | --- | --- | --- |
| None | 1 | 0.651 | 0.742 |
|  | 2 | 0.813 | 0.839 |
|  | 4 | 0.911 | 0.915 |
| Strong-positive | 1 | 0.509 | 0.679 |
|  | 2 | 0.570 | 0.723 |
|  | 4 | 0.601 | 0.749 |

Note: The attenuation factor does not exist when the maximum-likelihood discrepancy function is non-zero. The number of variables does not have an effect on the attenuation factor.

**Table S2.**

*Population value of the maximum-likelihood discrepancy function and RMSEA*

|  |  | *6 variables* | | *12 variables* | |
| --- | --- | --- | --- | --- | --- |
|  | thresholds | *λ* = 0.6 | *λ* = 0.9 | *λ* = 0.6 | *λ* = 0.9 |
| Mild and mixed | 1 | 0.004(0.021) | 0.416(0.215) | 0.018(0.018) | 1.327(0.157) |
|  | 2 | 0.006(0.025) | 0.374(0.204) | 0.024(0.021) | 1.205(0.149) |
|  | 4 | 0.005(0.024) | 0.363(0.201) | 0.022(0.020) | 1.171(0.147) |
| Strong and mixed | 1 | 0.018(0.045) | 0.730(0.285) | 0.073(0.037) | 2.276(0.205) |
|  | 2 | 0.023(0.050) | 0.825(0.303) | 0.090(0.041) | 2.537(0.217) |
|  | 4 | 0.024(0.052) | 0.882(0.313) | 0.095(0.042) | 2.694(0.223) |
| None +  Strong and mixed | 1 | 0.007(0.028) | 0.357(0.199) | 0.033(0.025) | 1.385(0.160) |
|  | 2 | 0.009(0.031) | 0.402(0.211) | 0.041(0.028) | 1.426(0.162) |
|  | 4 | 0.010(0.033) | 0.444(0.222) | 0.044(0.028) | 1.523(0.168) |

Note: The maximum-likelihood discrepancy function is zero in the conditions of no skewness and strong positive skewness. The population RMSEA appears in brackets.

**APPENDIX B. ANOVA for the logit of the *p*-value for chi-square under the study conditions**

**Table S3.**

*Results for the FA model*

|  |  | *p* = 6 variables | | | *p* = 12 variables | | |
| --- | --- | --- | --- | --- | --- | --- | --- |
| Condition | *df* | *F* | *p-value* |  | *F* | *p-value* |  |
| *λ* | 1 | 85016.3 | < .001 | .59 | 42177.2 | < .001 | .41 |
| γ | 4 | 8765.7 | < .001 | .37 | 9173.4 | < .001 | .38 |
| *N* | 1 | 1640.2 | < .001 | .02 | 4764.5 | < .001 | .07 |
| *τ* | 2 | 88.5 | < .001 | .00 | 302.3 | < .001 | .00 |
| *λ* × γ | 4 | 5561.1 | < .001 | .27 | 1480.4 | < .001 | .09 |
| *λ* × *N* | 1 | 1143.7 | < .001 | .02 | 4663.0 | < .001 | .07 |
| *γ* × *N* | 4 | 350.6 | < .001 | .02 | 702.1 | < .001 | .05 |
| * *×*τ* | 2 | 293.8 | < .001 | .00 | 590.3 | < .001 | .02 |
| *γ *×*τ* | 8 | 126.2 | < .001 | .02 | 542.5 | < .001 | .07 |
| *N *×*τ* | 2 | 1.8 | .17 | .00 | 7.7 | < .001 | .00 |
| *λ* × γ × *N* | 4 | 350.4 | < .001 | .02 | 731.5 | < .001 | .05 |
| *λ* × γ × *τ* | 8 | 54.5 | < .001 | .00 | 274.4 | < .001 | .04 |
| *λ* × *N *× *τ* | 2 | 9.6 | < .001 | .00 | 3.8 | .02 | .00 |
| *γ* × *N *× *τ* | 8 | 5.0 | < .001 | .00 | 3.7 | < .001 | .00 |
| *λ* × γ × *N *× *τ* | 8 | 3.5 | < .001 | .00 | 3.8 | < .001 | .00 |

*Note*. *p* is the number of variables, *λ* refers to the magnitude of the factor loadings, γ is Skewness, *N* is sample size, and *τ* is the number of thresholds (i.e., the number of response alternatives minus one).

**Table S4.**

*Results for the FAC model*

|  |  | *p* = 6 variables | | | *p* = 12 variables | | |
| --- | --- | --- | --- | --- | --- | --- | --- |
| Condition | *df* | *F* | *p-value* |  | *F* | *p-value* |  |
| *λ* | 1 | 7307.8 | < .001 | .11 | 3758.2 | < .001 | .06 |
| γ | 4 | 7324.4 | < .001 | .33 | 3994.3 | < .001 | .21 |
| *N* | 1 | 54.6 | < .001 | .00 | .17 | .68 | .00 |
| *τ* | 2 | 3030.5 | < .001 | .09 | 2422.3 | < .001 | .07 |
| *λ* × γ | 4 | 1433.5 | < .001 | .09 | 3241.7 | < .001 | .18 |
| *λ* × *N* | 1 | 1.1 | .29 | .00 | 1.9 | .16 | .00 |
| *γ* × *N* | 4 | 2.3 | .06 | .00 | 0.1 | .97 | .00 |
| * *×*τ* | 2 | 450.1 | < .001 | .01 | 1860.2 | < .001 | .06 |
| *γ *×*τ* | 8 | 994.3 | < .001 | .12 | 2052.4 | < .001 | .22 |
| *N *×*τ* | 2 | 14.4 | < .001 | .00 | 2.6 | .07 | .00 |
| *λ* × γ × *N* | 4 | 22.2 | < .001 | .00 | 3.6 | .01 | .00 |
| *λ* × γ × *τ* | 8 | 124.7 | < .001 | .02 | 1541.4 | < .001 | .17 |
| *λ* × *N *× *τ* | 2 | 0.1 | .90 | .00 | 0.6 | .54 | .00 |
| *γ* × *N *× *τ* | 8 | 2.3 | .02 | .00 | 2.0 | .04 | .00 |
| *λ* × γ × *N *× *τ* | 8 | 1.5 | .15 | .00 | 1.1 | .35 | .00 |

*Note*. *p* is the number of variables, *λ* refers to the magnitude of the factor loadings, γ is Skewness, *N* is sample size, and *τ* is the number of thresholds (i.e., the number of response alternatives minus one).

**Table S5.**

*Results for the WLSMV estimation of the FAC model*

|  |  | *p* = 6 variables | | | *p* = 12 variables | | |
| --- | --- | --- | --- | --- | --- | --- | --- |
| Condition | *df* | *F* | *p-value* |  | *F* | *p-value* |  |
| *λ* | 1 | 1506.8 | < .001 | .03 | 1050.6 | < .001 | .02 |
| γ | 4 | 78.0 | < .001 | .00 | 654.3 | < .001 | .05 |
| *N* | 1 | 22.3 | < .001 | .00 | 3401.3 | < .001 | .07 |
| *τ* | 2 | 36.2 | < .001 | .00 | 706.4 | < .001 | .03 |
| *λ* × γ | 4 | 152.0 | < .001 | .01 | 1618.4 | < .001 | .12 |
| *λ* × *N* | 1 | 532.9 | < .001 | .00 | 1915.4 | < .001 | .04 |
| *γ* × *N* | 4 | 21.7 | < .001 | .00 | 233.2 | < .001 | .02 |
| * *×*τ* | 2 | 170.5 | < .001 | .00 | 1096.3 | < .001 | .04 |
| *γ *×*τ* | 8 | 111.8 | < .001 | .02 | 454.7 | < .001 | .07 |
| *N *×*τ* | 2 | 101.7 | < .001 | .00 | 154.9 | < .001 | .00 |
| *λ* × γ × *N* | 4 | 363.5 | < .001 | .03 | 1308.6 | < .001 | .10 |
| *λ* × γ × *τ* | 8 | 121.4 | < .001 | .02 | 541.7 | < .001 | .08 |
| *λ* × *N *× *τ* | 2 | 136.2 | < .001 | .00 | 386.3 | < .001 | .02 |
| *γ* × *N *× *τ* | 8 | 56.7 | < .001 | .00 | 121.2 | < .001 | .02 |
| *λ* × γ × *N *× *τ* | 8 | 16.2 | < .001 | .00 | 94.9 | < .001 | .02 |

*Note*. *p* is the number of variables, *λ* refers to the magnitude of the factor loadings, γ is Skewness, *N* is sample size, and *τ* is the number of thresholds (i.e., the number of response alternatives minus one).

**Table S6.**

*Results for the* d*T_M_ (Satorra-Bentler correction for the difference chi-square) statistic*

|  |  | *p* = 6 variables | | | *p* = 12 variables | | |
| --- | --- | --- | --- | --- | --- | --- | --- |
| Condition | *df* | *F* | *p-value* |  | *F* | *p-value* |  |
| *λ* | 1 | 1245.9 | < .001 | .02 | 14298.7 | < .001 | .23 |
| γ | 4 | 321.5 | < .001 | .02 | 2556.4 | < .001 | .18 |
| *N* | 1 | 179.8 | < .001 | .00 | 3843.2 | < .001 | .07 |
| *τ* | 2 | 8.2 | < .001 | .00 | 3475.9 | < .001 | .13 |
| *λ* × γ | 4 | 144.9 | < .001 | .01 | 1354.9 | < .001 | .10 |
| *λ* × *N* | 1 | 496.3 | < .001 | .00 | 624.0 | < .001 | .01 |
| *γ* × *N* | 4 | 9.8 | < .001 | .00 | 729.9 | < .001 | .06 |
| * *×*τ* | 2 | 131.8 | < .001 | .00 | 1273.6 | < .001 | .05 |
| *γ *×*τ* | 8 | 131.5 | < .001 | .02 | 573.4 | < .001 | .09 |
| *N *×*τ* | 2 | 132.1 | < .001 | .00 | 85.0 | < .001 | .00 |
| *λ* × γ × *N* | 4 | 432.7 | < .001 | .03 | 3242.8 | < .001 | .21 |
| *λ* × γ × *τ* | 8 | 140.8 | < .001 | .02 | 467.3 | < .001 | .07 |
| *λ* × *N *× *τ* | 2 | 149.7 | < .001 | .00 | 387.1 | < .001 | .02 |
| *γ* × *N *× *τ* | 8 | 54.6 | < .001 | .00 | 132.4 | < .001 | .02 |
| *λ* × γ × *N *× *τ* | 8 | 20.4 | < .001 | .00 | 490.4 | < .001 | .08 |

*Note*. *p* is the number of variables, *λ* refers to the magnitude of the factor loadings, γ is skewness, *N* is sample size, and *τ* is the number of thresholds (i.e., the number of response alternatives minus one).

**Table S7.**

*Results for the GRM model*

|  |  | *p* = 6 variables | | | *p* = 12 variables | | |
| --- | --- | --- | --- | --- | --- | --- | --- |
| Condition | *df* | *F* | *p-value* |  | *F* | *p-value* |  |
| *λ* | 1 | 2320.6 | < .001 | .04 | 102.6 | < .001 | .00 |
| γ | 4 | 2291.5 | < .001 | .13 | 339.4 | < .001 | .02 |
| *N* | 1 | 710.2 | < .001 | .01 | 82.2 | < .001 | .00 |
| *τ* | 2 | 1235.5 | < .001 | .04 | 481.3 | < .001 | .02 |
| *λ* × γ | 4 | 2129.0 | < .001 | .12 | 285.5 | < .001 | .02 |
| *λ* × *N* | 1 | 19.6 | < .001 | .00 | 80.6 | < .001 | .00 |
| *γ* × *N* | 4 | 128.6 | < .001 | .00 | 8.0 | < .001 | .00 |
| * *×*τ* | 2 | 169.5 | < .001 | .00 | 0.8 | .47 | .00 |
| *γ *×*τ* | 8 | 140.2 | < .001 | .02 | 19.1 | < .001 | .00 |
| *N *×*τ* | 2 | 11.4 | < .001 | .00 | 4.9 | < .001 | .00 |
| *λ* × γ × *N* | 4 | 61.4 | < .001 | .00 | 5.9 | < .001 | .00 |
| *λ* × γ × *τ* | 8 | 131.3 | < .001 | .02 | 8.1 | < .001 | .00 |
| *λ* × * *× *τ* | 2 | 14.9 | < .001 | .00 | 25.4 | < .001 | .00 |
| *γ* × *N *× *τ* | 8 | 26.8 | < .001 | .00 | 9.6 | < .001 | .00 |
| *λ* × γ × *N *× *τ* | 8 | 37.7 | < .001 | .00 | 8.3 | < .001 | .00 |

*Note*. *p* is the number of variables, *λ* refers to the magnitude of the factor loadings, γ is Skewness, *N* is sample size, and *τ* is the number of thresholds (i.e., the number of response alternatives minus one).

**APPENDIX C. Analysis of the *p*-value for the simulation study**

**Figure S1**. *p*-value for chi-square under the conditions manipulated in the simulation. FA model.
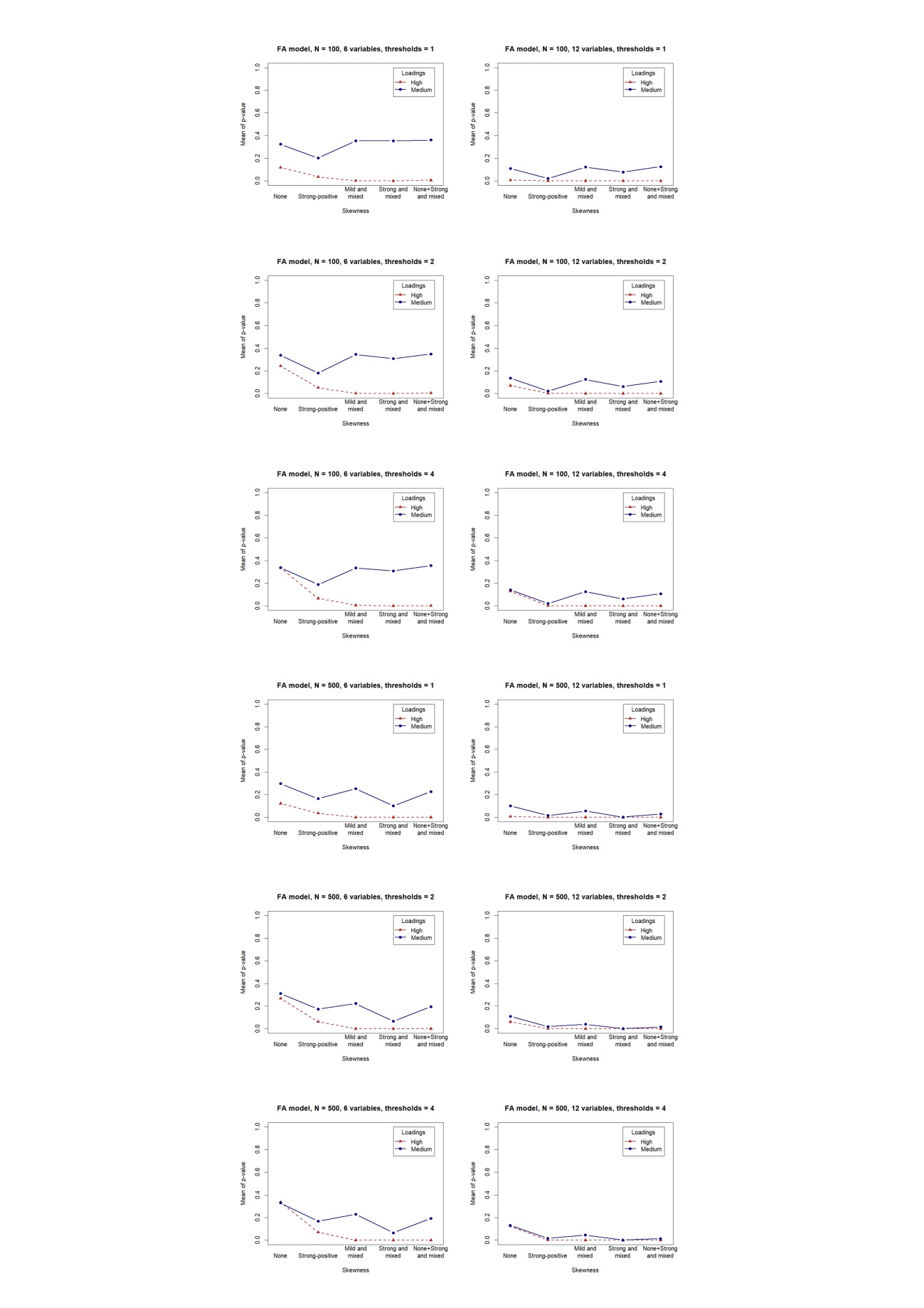


**Figure S2**. *p*-value for chi-square as under the conditions manipulated in the simulation. FAC model.
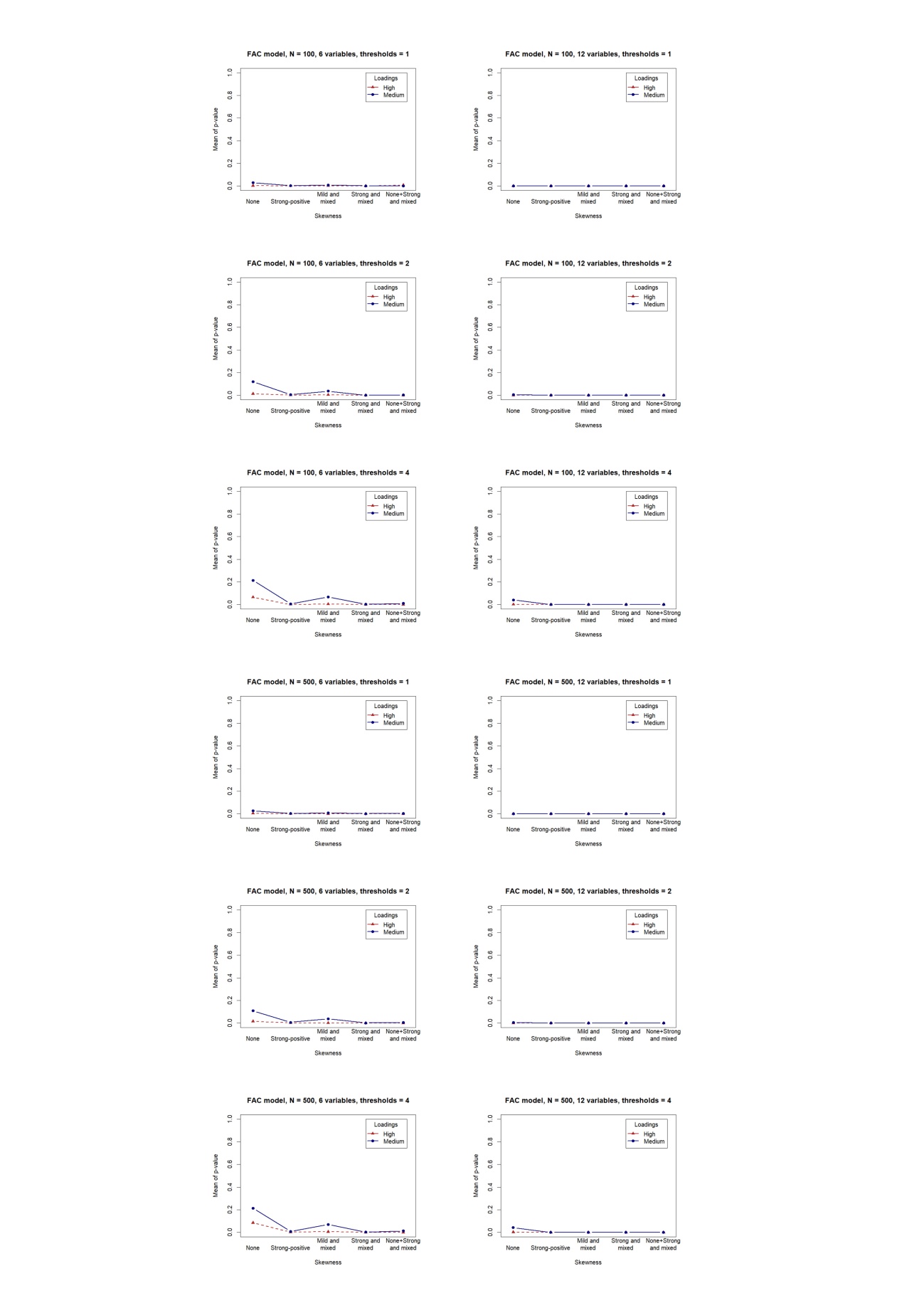


**Figure S3**. *p*-value for chi-square as a function of the conditions manipulated in the simulation. GRM model by Samejima.


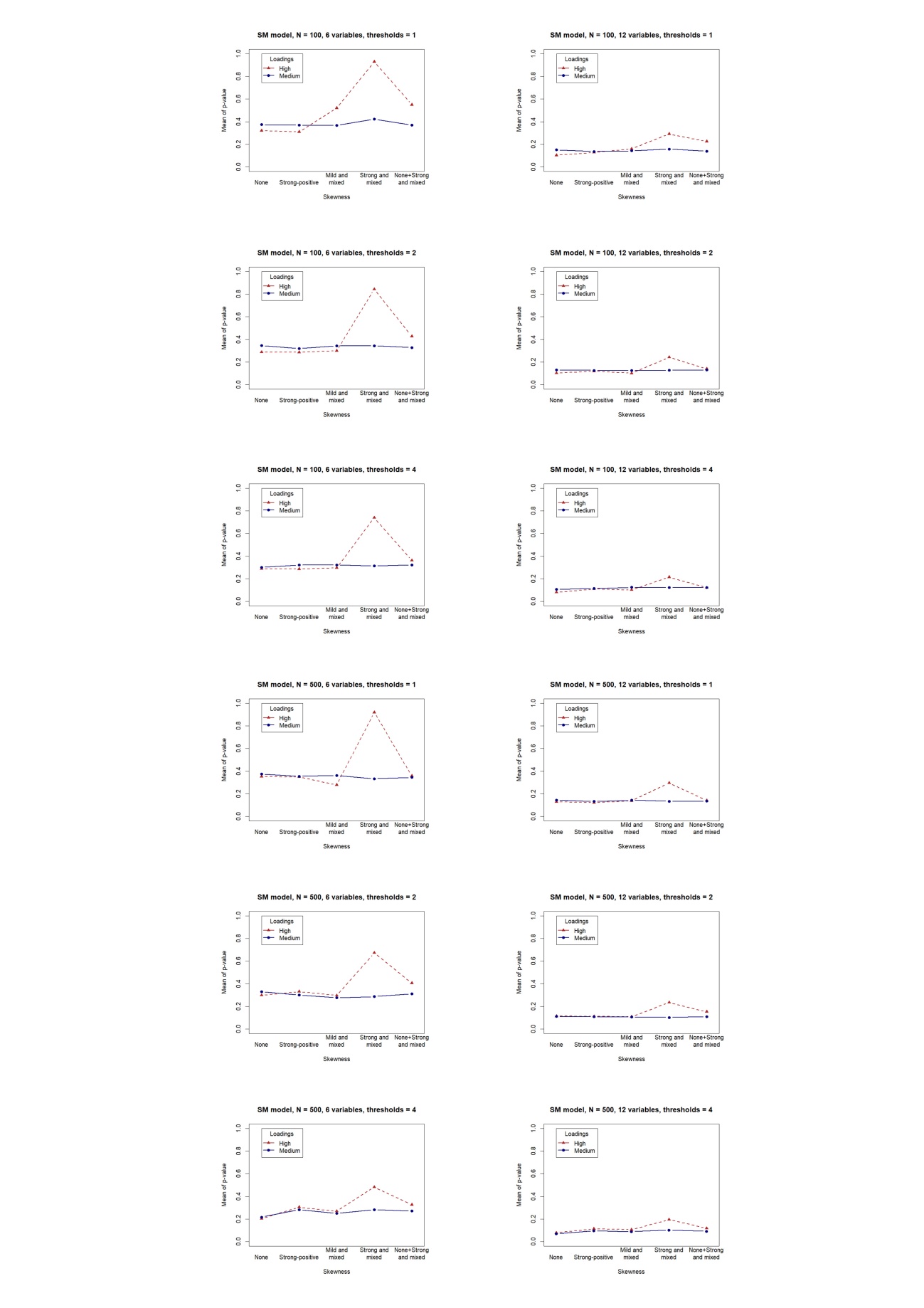


**APPENDIX D. Recovery of the parameters for the simulation study**

**Table S8.**

Recovery of lambda for the conditions manipulated in the simulation study

| Condition | Levels | FA | WLSMV | FAC | GRM |
| --- | --- | --- | --- | --- | --- |
| *N* | 100 | 0.22(0.28) | 0.11(0.16) | 0.12(0.17) | 0.09(0.06) |
|  | 500 | 0.24(0.51) | 0.10(0.60) | 0.10(0.42) | 0.04(0.03) |
| Variables | 6 | 0.26(0.55) | 0.08(0.10) | 0.10(0.17) | 0.07(0.06) |
|  | 12 | 0.20(0.18) | 0.13(0.65) | 0.12(0.42) | 0.06(0.04) |
| Thresholds | 1 | 0.23(0.09) | 0.08(0.07) | 0.09(0.08) | 0.08(0.06) |
|  | 2 | 0.20(0.16) | 0.07(0.08) | 0.08(0.09) | 0.07(0.059 |
|  | 4 | 0.27(0.68) | 0.17(0.79) | 0.16(0.54) | 0.06(0.05) |
| Loadings | .60 | 0.19(0.36) | 0.16(0.64) | 0.16(0.42) | 0.10(0.06) |
|  | .90 | 0.27(0.45) | 0.05(0.09) | 0.05(0.15) | 0.03(0.02) |
| Skewness | None | 0.09(0.04) | 0.05(0.04) | 0.05(0.04) | 0.05(0.04) |
|  | SP | 0.36(0.88) | 0.27(1.01) | 0.25(0.69) | 0.08(0.06) |
|  | MM | 0.16(0.05) | 0.06(0.04) | 0.06(0.05) | 0.06(0.04) |
|  | SM | 0.32(0.10) | 0.09(0.08) | 0.10(0.09) | 0.08(0.06) |
|  | N+SM | 0.23(0.06) | 0.07(0.06) | 0.09(0.08) | 0.07(0.05) |

Note: The table contains the mean and the (standard deviation) of RMSE.

**Table S9.**

Recovery of psi for the conditions manipulated in the simulation study

| Condition | Levels | FA | WLSMV | FAC | GRM |
| --- | --- | --- | --- | --- | --- |
| *N* | 100 | 0.20(0.26) | 0.11(0.12) | 0.13(0.21) | 0.12(0.05) |
|  | 500 | 0.21(0.39) | 0.07(0.32) | 0.08(0.33) | 0.05(0.02) |
| Variables | 6 | 0.22(0.44) | 0.09(0.15) | 0.13(0.37) | 0.09(0.06) |
|  | 12 | 0.19(0.15) | 0.09(0.31) | 0.09(0.14) | 0.08(0.04) |
| Thresholds | 1 | 0.20(0.11) | 0.08(0.05) | 0.11(0.08) | 0.10(0.06) |
|  | 2 | 0.18(0.13) | 0.08(0.08) | 0.09(0.09) | 0.08(0.05) |
|  | 4 | 0.23(0.55) | 0.11(0.42) | 0.12(0.47) | 0.07(0.04) |
| Loadings | .60 | 0.11(0.24) | 0.09(0.31) | 0.12(0.26) | 0.09(0.06) |
|  | .90 | 0.30(0.38) | 0.09(0.15) | 0.10(0.30) | 0.08(0.05) |
| Skewness | None | 0.10(0.06) | 0.05(0.03) | 0.05(0.03) | 0.06(0.03) |
|  | SP | 0.30(0.70) | 0.17(0.54) | 0.17(0.61) | 0.09(0.05) |
|  | MM | 0.17(0.09) | 0.06(0.04) | 0.07(0.05) | 0.07(0.04) |
|  | SM | 0.25(0.13) | 0.09(0.05) | 0.12(0.09) | 0.11(0.06) |
|  | N+SM | 0.21(0.11) | 0.08(0.04) | 0.012(0.09) | 0.09(0.05) |

Note: psi is the standard deviation of the errors. The table contains the mean and the (standard deviation) of RMSEA.

**Table S10.**

Recovery of tau under the conditions manipulated in the simulation study

| Condition | Levels | WLSMV | FAC | GRM |
| --- | --- | --- | --- | --- |
| *N* | 100 | 0.29(0.55) | 0.27(0.43) | 0.18(0.07) |
|  | 500 | 0.30(0.98) | 0.25(0.64) | 0.12(0.07) |
| Variables | 6 | 0.32(1.02) | 0.23(0.40) | 0.15(0.08) |
|  | 12 | 0.27(0.47) | 0.29(0.65) | 0.015(0.07) |
| Thresholds | 1 | 0.10(0.05) | 0.10(0.05) | 0.10(0.06) |
|  | 2 | 0.14(0.08) | 0.14(0.08) | 0.16(0.07) |
|  | 4 | 0.64(1.30) | 0.54(0.87) | 0.18(0.08) |
| Loadings | .60 | 0.28(0.59) | 0.29(0.67) | 0.16(0.08) |
|  | .90 | 0.31(0.95) | 0.23(0.38) | 0.13(0.07) |
| Skewness | None | 0.10(0.05) | 0.10(0.05) | 0.11(0.06) |
|  | SP | 0.77(1.56) | 0.69(1.05) | 0.17(0.08) |
|  | MM | 0.17(0.26) | 0.15(0.16) | 0.14(0.08) |
|  | SM | 0.26(0.54) | 0.20(0.27) | 0.17(0.08) |
|  | N+SM | 0.17(0.28) | 0.17(0.20) | 0.15(0.07) |

Note: The table contains the mean and the (standard deviation) of RMSE.

**Figure S4**. Recovery of lambda as related to true lambda, skewness, sample size, and method.

**
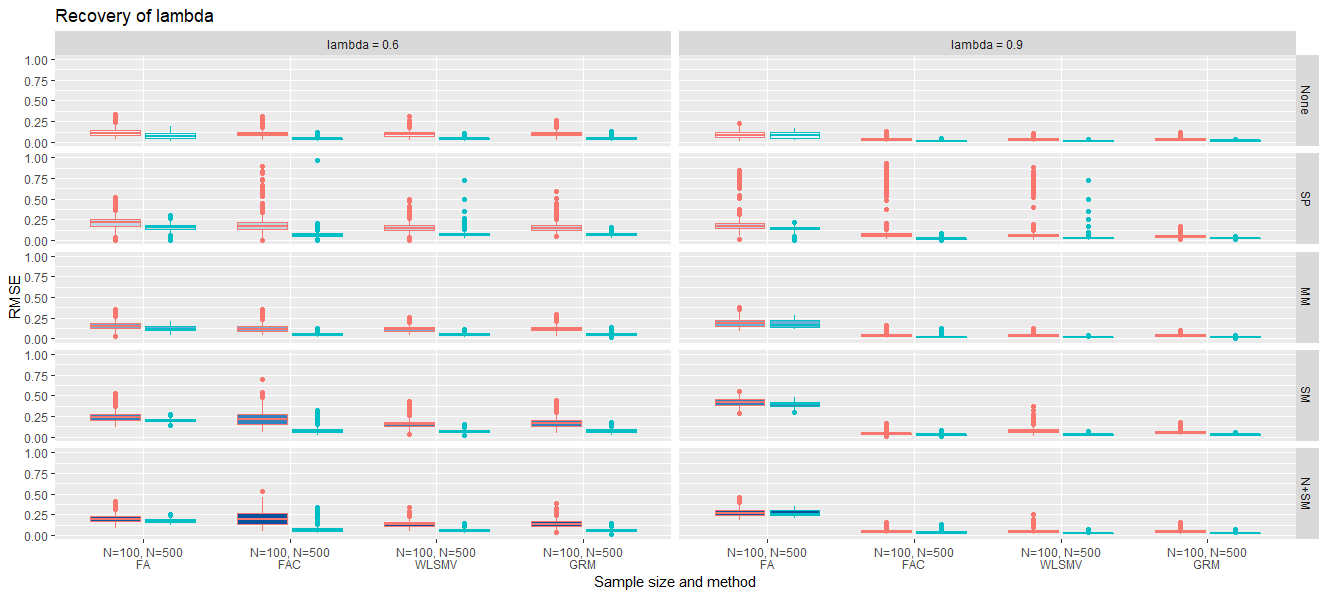
**

**Figure S5**. Recovery of lambda as related to number of thresholds, skewness, sample size, and method.

**
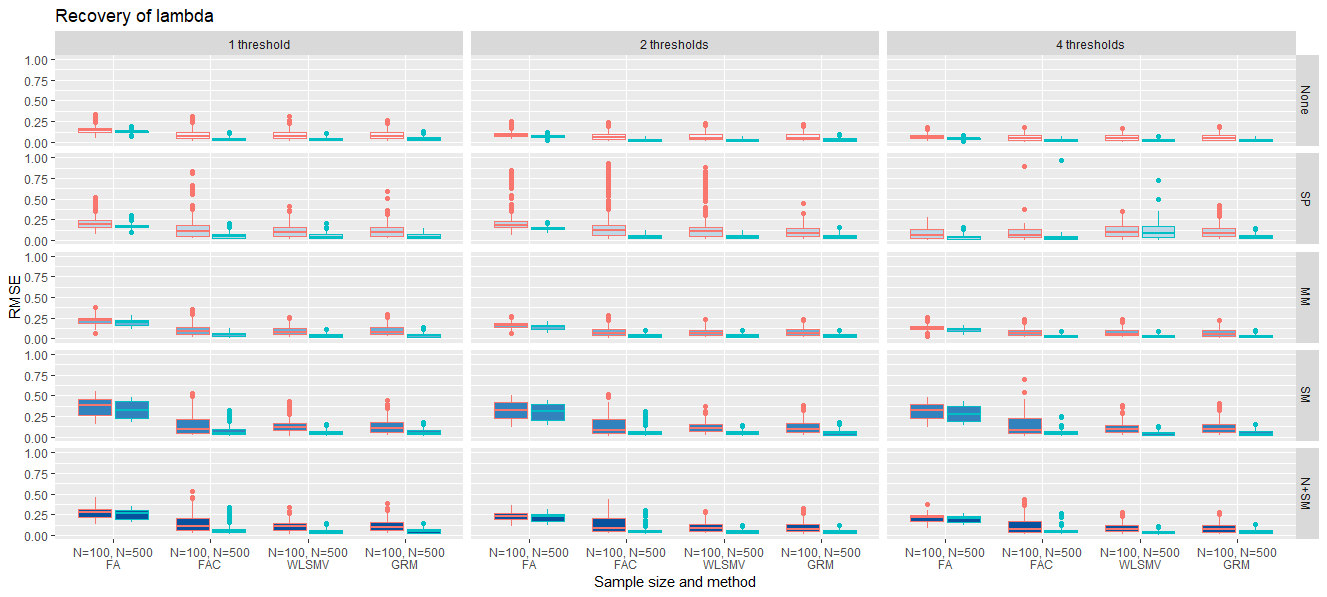
**

**Figure S6**. Recovery of lambda as related to number of variables, skewness, sample size, and method.

**
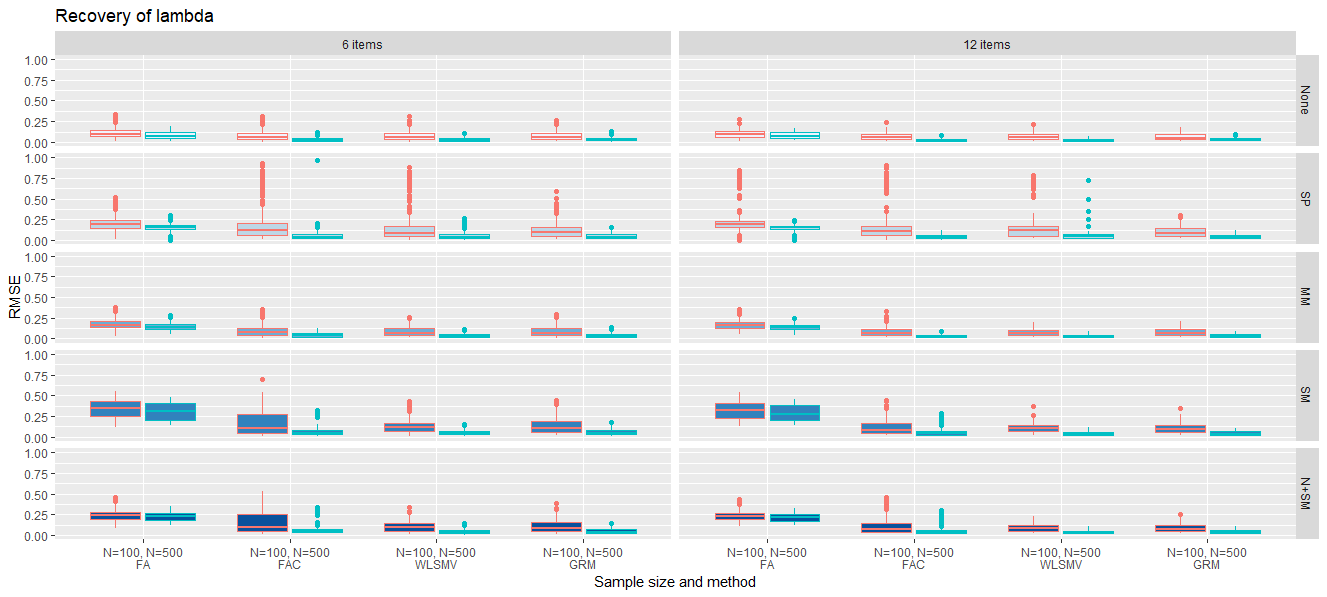
**

**Figure S7**. Recovery of tau as related to true lambda, skewness, sample size, and method.

**
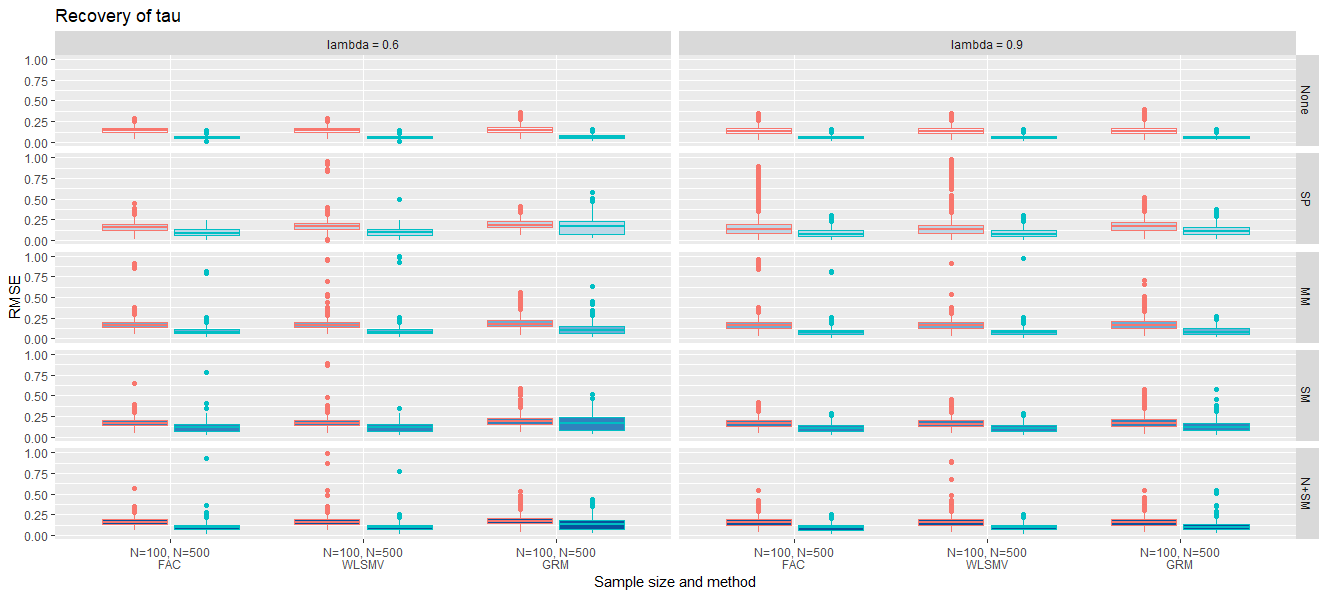
**

**Figure S8**. Recovery of tau as related to number of thresholds, skewness, sample size, and method.

**
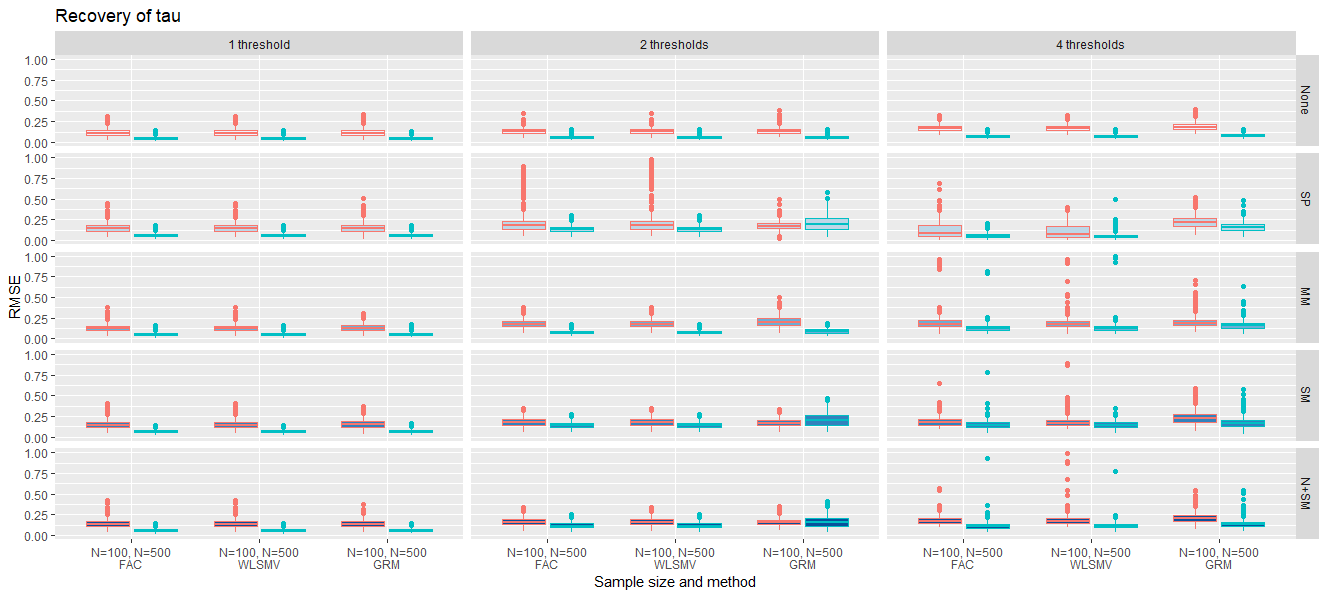
**

**Figure S9**. Recovery of tau as related to number of variables, skewness, sample size, and method.

**
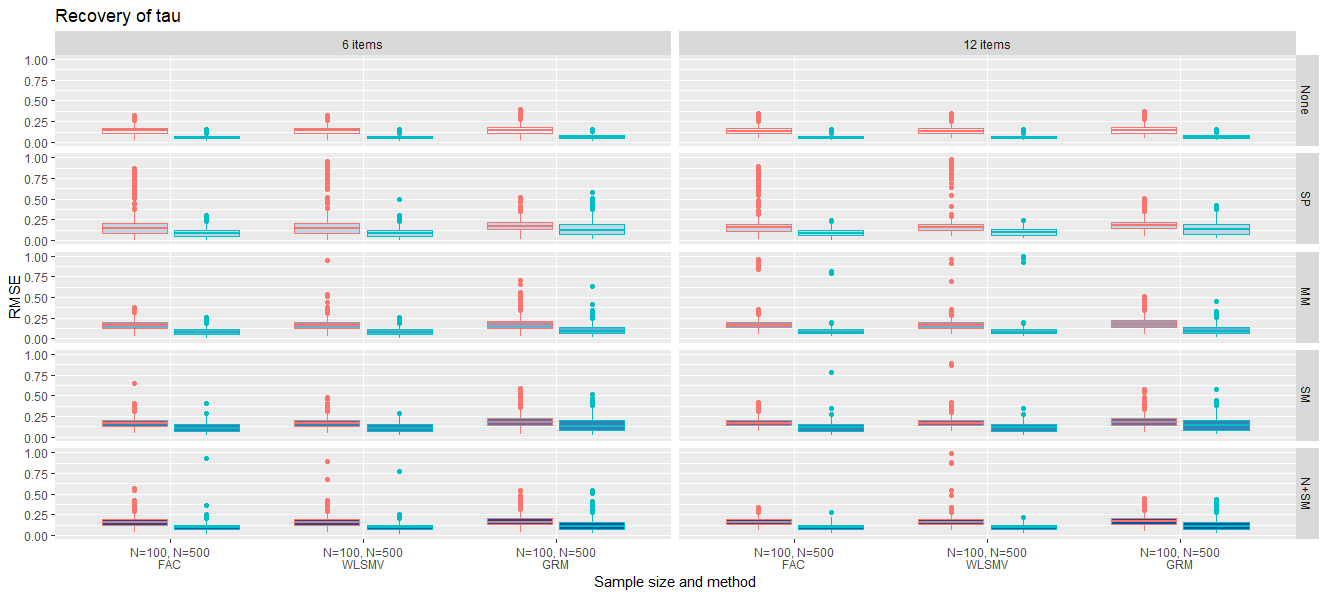
**

**APPENDIX E. R code to generate thresholds from gamma**

# Simulate a categorical variable that follows a factor model and has

# a prescribed skewness.

# Based on Olsson(!979), equation (7)

# Input variables:

# lambda : factor loadings

# gamma : coefficient of skewness

# nThreshols : number of thresholds (categories minus one`

# N : sample size

# Output: list with elements:

# tau : thresholds

# y : simulated categorical variable

# generate thresholds for the given gamma, lambda,

# number of variables and number of thresholds

generateTau <- function(gamma,lambda,nThresholds){

tau <- rep(NA,nThresholds)

# Solution to Olsson (1979), eq. (7)

p <- 0.5 - gamma*sqrt(nThresholds/(16+4*gamma^2*nThresholds))

# c.d.f. for the binomial(nThresholds,p) distribution

probs <- pbinom(0:nThresholds,nThresholds,p)

# transform probs to standard normal deviates

tau <- qnorm(probs)[1:nThresholds]

return(tau)

}

# function to simulate item responses

simulate.y <- function(lambda,gamma,tau,xi){

psi <- sqrt(1-lambda^2)

y.star <- rnorm(N,lambda*xi,psi)

y <- cut(y.star,c(-Inf,tau,Inf),labels=F)

return(y = y)

}

# Example of how to generate the thresholds in Table 3

# input data

N <- 500

lambda <- 0.8

gamma <- 2

nThresholds <- 4

# generate thresholds

tau <- generateTau(gamma = gamma,

lambda = lambda,

nThresholds = nThresholds)

# simulate factor scores

xi <- rnorm(N, mean = 0, sd=1)

# simulate responses

y <- simulate.y(lambda=lambda,gamma=gamma,tau=tau,xi=xi)

cat(sprintf(" true gamma : %.2f\n",gamma))

cat(" threshold values: ")

cat(sprintf("%.2f ",tau))

cat("\n")

cat(sprintf(" empirical gamma : %.2f\n",e1071::skewness(y,type=1)))

cat("\n")

**APPENDIX F. R code for estimating the models in the simulation study**

library(psych)

library(lavaan)

library(mirt)

# Note: this script factor-analyzes a data matrix named x using the procedures employed in the simulation study

nVars <- ncol(x)

# Parallel analysis

fa.parallel(x,cor="cor",plot=F) # PA based on Pearson correlations

fa.parallel(x,cor="poly",correct=0,plot=F) # PA based on polychoric correlations

# FA model, chi square and parameter estimates

fa1 <- fa(x,nfactors=1,fm="ml",cor="cor",plot=F)

fa2 <- fa(x,nfactors=1,fm="ml",cor="cor",plot=F,rotate="none")

anova(fa1,fa2)

fa.lambda.est <- as.vector(fa1$loadings)

fa.psi.est <- sqrt( as.vector(fa1$uniquenesses) )

# FAC model

fac1 <- fa(x,nfactors=1,fm="ml",cor="poly",correct=0,plot=F)

fac2 <- fa(x,nfactors=2,fm="ml",cor="poly",correct=0,plot=F,rotate="none")

anova(fac1,fac2)

fac.lambda.est <- as.vector(fa1$loadings)

fac.psi.est <- sqrt( as.vector(fa1$uniquenesses) )

if(sessionInfo()$otherPkgs$psych$Version<"2"){

polychoric(x)$tau # Do not use versions of psych above 1.9.12 to run this line

}

# WLSMV estimation

cx <- ' =~ '

for(i in 1:nVars){

cx <- paste(cx,sprintf('x%1.0d',i),sep='')

if(i < nVars){ cx <- paste(cx,' + ',sep='') }

}

f1 <- paste('efa(\"efa\")*f1',cx,sep='')

f2 <- paste('efa(\"efa\")*f1 + efa("efa")*f2',cx,sep='')

colnames(x) <- sprintf("x%d",1:nVars)

efa_f1 <- cfa(model=f1,data=x,estimator = "WLSMV",ordered = TRUE)

efa_f2 <- cfa(model=f2,data=x,estimator = "WLSMV",ordered = TRUE)

sb <- lavTestLRT(efa_f1, efa_f2, method = "satorra.bentler.2010")

lrG2 <- sb$Chisq[2]-sb$Chisq[1]

# likelihood ratio chi-square for WLSMV

dfG2 <- sb$Df[2]-sb$Df[1]

pG2 <- pchisq(lrG2,dfG2,lower.tail = F)

dTM <- sb$`Chisq diff`[2]

# Satorra-Bentler difference between chi-square values

dTMF.df <- sb$`Df diff`[2]

dTM.p <- sb$`Pr(>Chisq)`[2]

# parameter estimates for WLSMV

parTable(efa_f1)

# GRM

grm1 <- mirt(x,model=1,itemtype = 'graded',verbose=F,technical=list(NCYCLES=500))

grm2 <- mirt(x,model=2,itemtype = 'graded',verbose=F,technical=list(NCYCLES=500))

# chi-square for GRM

anova(grm1,grm2)

# Convert IRT to factor analysis parameters Paek et al. (2018)

irt.to.fa <- function(m,nVars,nThresholds){

f <- coef(m,as.data.frame=T,complete=T)

np <- nVars*(nThresholds+1)

lambda.est <- rep(NA,nVars)

psi.est <- rep(NA,nVars)

tau.est <- rep(NA,nVars*nThresholds)

k <- 0

for(i in 1:nVars){

den <- sqrt(1.7^2 + coef(m)[[i]][1]^2)

lambda.est[i] <- coef(m)[[i]][1] / den

psi.est[i] <- sqrt( 1 - lambda.est[i]^2 )

for(j in 2:length(coef(m)[[i]])){

k <- k+1

tau.est[k] <- - coef(m)[[i]][j]/den

}

}

return(list(lambda.est=lambda.est,

psi.est=psi.est, tau.est=tau.est))

}

irt.to.fa(grm1,nVars,nThresholds=3)

**APPENDIX G. R code for the empirical application**

# read the data from the Georgia Tech. Psychometric Research and Development Lab

data <- read.fwf(url("https://ggum.gatech.edu/cendat.txt"),widths = c(5,rep(1,20)))[,2:21]

############## print descriptive statistics #######################

cat("\n")

cat("\n")

cat("Descriptive statistics:\n")

for(i in 1:ncol(data)){

cat(sprintf("Variable %2.0f. (freqs) = ",i))

tb <- table(data[,i])

for(j in 1:6){

cat(sprintf(" %3.0f",tb[j]))

}

cat(sprintf(" (mean,sd,skewness,kurtosis) = %6.2f %6.2f %6.2f %6.2f\n",

mean(data[,i]),

sd(data[,i]),

e1071::skewness(data[,i],na.rm=T),

e1071::kurtosis(data[,i],na.rm=T)))

}

cat("\n")

cat("\n")

library(semTools)

print(mardiaSkew(data)) # multivariate skew

print(mardiaKurtosis(data)) # multivariate kurtosis

############## print descriptive statistics. ENDS #################

############## parallel analysis ##################################

library(psych)

cat("Running parallel analysis (FA model):\n")

retPA <- fa.parallel(data,cor="cor",plot=T)

cat("Running parallel analysis (FA model):\n")

retPAC <- fa.parallel(data,cor="poly",plot=T)

cat("\n")

############## parallel analysis. ENDS#############################

######### check for underlying normality of latent responses ######

library(discnorm)

pvalue <- bootTest(data, B=500)

print(pvalue)

######### check for underlying normality. ENDS ####################

# Prepare the models and the data to run lavaan ##################

library(lavaan)

nItems <- ncol(data)

colnames(data) <- sprintf("y%.0f",1:nItems)

cy <- ' =~ '

for(i in 1:nItems){

cy <- paste(cy,sprintf('y%1.0d',i),sep='')

if(i < nItems){ cy <- paste(cy,' + ',sep='') }

}

f1 <- paste('efa(\"efa\")*f1',cy,sep='')

f2 <- paste('efa(\"efa\")*f1 + efa("efa")*f2',cy,sep='')

f3 <- paste('efa(\"efa\")*f1 + efa(\"efa\")*f2 + efa("efa")*f3',cy,sep='')

f4 <- paste('efa(\"efa\")*f1 + efa(\"efa\")*f2 + efa("efa")*f3 + efa("efa")*f4',cy,sep='')

f5 <- paste('efa(\"efa\")*f1 + efa(\"efa\")*f2 + efa("efa")*f3 + efa("efa")*f4 + efa("efa")*f5',cy,sep='')

f6 <- paste('efa(\"efa\")*f1 + efa(\"efa\")*f2 + efa("efa")*f3 + efa("efa")*f4 + efa("efa")*f5 + efa("efa")*f6',cy,sep='')

f7 <- paste('efa(\"efa\")*f1 + efa(\"efa\")*f2 + efa("efa")*f3 + efa("efa")*f4 + efa("efa")*f5 + efa("efa")*f6 + efa("efa")*f7',cy,sep='')

# Prepare the models and the data to run lavaan. ENDS ############

###### Results in Table 10 #######################################

# Fit FA, includes chi square and mean corrected chi-square by Satorra and Bentler (TM)

lav1m <- cfa(model = f1,data = data,estimator = "MLM")

lav2m <- cfa(model = f2,data = data,estimator = "MLM")

lav3m <- cfa(model = f3,data = data,estimator = "MLM")

lav4m <- cfa(model = f4,data = data,estimator = "MLM")

print(lav1m) # show chi2 and TM for the 1-factor model

# mean and variance corrected statistic by Asparouhov and Muthen (TMV)

lav1mv <- cfa(model = f1,data = data,estimator = "MLMV")

lav2mv <- cfa(model = f2,data = data,estimator = "MLMV")

lav3mv <- cfa(model = f3,data = data,estimator = "MLMV")

lav4mv <- cfa(model = f4,data = data,estimator = "MLMV")

print(lav1mv) # show chi2 and TMV for the 1-factor model

###### Results in Table 10. ENDS####################################

###### Results in Table 11 #######################################

# fit categorical models in lavaan using WLSMV estimator and

# a first order (mean) correction of chi-square. TM by Satorra-Bentler

lav1r <- cfa(model = f1,data = data,estimator = "WLSMV",ordered = TRUE,test = "Satorra-Bentler")

lav2r <- cfa(model = f2,data = data,estimator = "WLSMV",ordered = TRUE,test = "Satorra-Bentler")

lav3r <- cfa(model = f3,data = data,estimator = "WLSMV",ordered = TRUE,test = "Satorra-Bentler")

lav4r <- cfa(model = f4,data = data,estimator = "WLSMV",ordered = TRUE,test = "Satorra-Bentler")

lav5r <- cfa(model = f5,data = data,estimator = "WLSMV",ordered = TRUE,test = "Satorra-Bentler")

lav6r <- cfa(model = f6,data = data,estimator = "WLSMV",ordered = TRUE,test = "Satorra-Bentler")

lav7r <- cfa(model = f7,data = data,estimator = "WLSMV",ordered = TRUE,test = "Satorra-Bentler")

print(lav1r) # show G2 and TM for the 1-factor model

# fit categorical models in lavaan using WLSMV estimator and a second

# order (mean and variance) correction of chi-square. TMV by Asparouhov and Muthen

lav1 <- cfa(model = f1,data = data,estimator = "WLSMV",ordered = TRUE)

lav2 <- cfa(model = f2,data = data,estimator = "WLSMV",ordered = TRUE)

lav3 <- cfa(model = f3,data = data,estimator = "WLSMV",ordered = TRUE)

lav4 <- cfa(model = f4,data = data,estimator = "WLSMV",ordered = TRUE)

lav5 <- cfa(model = f5,data = data,estimator = "WLSMV",ordered = TRUE)

lav6 <- cfa(model = f6,data = data,estimator = "WLSMV",ordered = TRUE)

lav7 <- cfa(model = f7,data = data,estimator = "WLSMV",ordered = TRUE)

print(lav1) # show G2 and TMV for the 1-factor model

# compute the Satorra-Bentler statistic for pairs of nested models (dTM)

lav_12 <- lavTestLRT(lav1, lav2, method = "satorra.bentler.2010")

lav_23 <- lavTestLRT(lav2, lav3, method = "satorra.bentler.2010")

lav_34 <- lavTestLRT(lav3, lav4, method = "satorra.bentler.2010")

lav_45 <- lavTestLRT(lav4, lav5, method = "satorra.bentler.2010")

lav_56 <- lavTestLRT(lav5, lav6, method = "satorra.bentler.2010")

lav_67 <- lavTestLRT(lav6, lav7, method = "satorra.bentler.2010")

print(lav_12) # show Td to compare the 1 and 2 factor models

###### Results in Table 11. ENDS##################################

###### Results in Table 12 #######################################

library(psych)

# Estimate the FAC model using polychoric correlations

cfa1 = fa(data,1, cor="poly", fm="ml")

cfa2 = fa(data,2, cor="poly", fm="ml")

cfa3 = fa(data,3, cor="poly", fm="ml")

cfa4 = fa(data,4, cor="poly", fm="ml")

cfa5 = fa(data,5, cor="poly", fm="ml")

cfa6 = fa(data,6, cor="poly", fm="ml")

cfa7 = fa(data,7, cor="poly", fm="ml")

print(anova(cfa1,cfa2,cfa3,cfa4,cfa5,cfa6,cfa7))

###### Results in Table 12. ENDS #################################

###### Results in Table 13 #######################################

library(mirt)

# "Estimating the GRM model

grm1 <- mirt(data, model=1, itemtype = 'graded', method='EM')

grm2 <- mirt(data, model=2, itemtype = 'graded', method='EM')

grm3 <- mirt(data, model=3, itemtype = 'graded', method='EM')

grm4 <- mirt(data, model=4, itemtype = 'graded', method='EM')

grm5 <- mirt(data, model=5, itemtype = 'graded', method='EM')

# chi-square for SM

print(anova(grm1,grm2,grm3,grm4,grm5))

# Estimating the MNCM model:

mncm1 <- mirt(data,model=1,itemtype = 'nominal',method='EM')

mncm2 <- mirt(data,model=2,itemtype = 'nominal',method='EM')

mncm3 <- mirt(data,model=3,itemtype = 'nominal',method='EM')

mncm4 <- mirt(data,model=4,itemtype = 'nominal',method='EM')

mncm5 <- mirt(data,model=5,itemtype = 'nominal',method='EM')

# chi-square for MNCM:

print(anova(mncm1,mncm2,mncm3,mncm4,mncm5))

# chi-square for GRM agains the MNCM:

anova(mncm1,grm1)

anova(mncm2,grm2)

anova(mncm3,grm3)

anova(mncm4,grm4)

anova(mncm5,grm5)

###### Results in Table 13. ENDS #################################

###### Analysis of normality for latent scores ####################

# Estimating the SM-IRT model under normal factor scores

sms1 <- mirt(data, model=1, itemtype = 'graded', dentype='Gaussian',verbose=F, technical=list(NCYCLES=1000))

# Estimating Davidian curves for factor scores

sms2 <- mirt(data, model=1, itemtype = 'graded', dentype='Davidian-2',verbose=F, technical=list(NCYCLES=1000))

sms3 <- mirt(data, model=1, itemtype = 'graded', dentype='Davidian-3',verbose=F, technical=list(NCYCLES=1000))

# Estimating empirical histogram for factor scores

sms4 <- mirt(data, model=1, itemtype = 'graded', dentype='empiricalhist',verbose=F, technical=list(NCYCLES=1000))

# Compare fit

anova(sms1,sms2,sms3,sms4)

# Plotting factor scores

plot(sms1,type='Normal')

plot(sms2, type = 'Davidian') # shape of latent trait distribution

plot(sms3, type = 'Davidian') # shape of latent trait distribution

plot(sms4, type = 'empiricalhist') # shape of latent trait distribution

coef(sms1, simplify=TRUE)

coef(sms2, simplify=TRUE)

coef(sms3, simplify=TRUE)

# Dispersion plots for factor scores and correlations

fs2.n <- fscores(sms2) # assume normal prior

fs2.d2 <- fscores(sms2, use_dentype_estimate=TRUE) # use Davidian-2 estimated prior shape

fs2.d3 <- fscores(sms3, use_dentype_estimate=TRUE) # use Davidian-3 estimated prior shape

fs2.e <- fscores(sms3, use_dentype_estimate=TRUE) # use empirical histogram

plot(fs2.n,fs2.d2,

xlab="normal factor score",

ylab="Davidian-2 factor score",

main=sprintf("correlation = %.2f",cor(fs2.n,fs2.d2)))

plot(fs2.n,fs2.d3,

xlab="normal factor score",

ylab="Davidian-3 factor score",

main=sprintf("correlation = %.2f",cor(fs2.n,fs2.d3)))

plot(fs2.n,fs2.e,

xlab="normal factor score",

ylab="Empirical histogram factor score",

main=sprintf("correlation = %.2f",cor(fs2.n,fs2.e)))

# Item fit for the different models

itemfit(sms1) # assume normal prior

itemfit(sms2) # assume Davidian-2 prior

itemfit(sms3) # assume Davidian-3 prior

itemfit(sms4) # assume empirical histogram

###### Analysis of normality for latent scores. ENDS ################
